# The proteomic complexity and rise of the primordial ancestor of diversified life

**DOI:** 10.1186/1471-2148-11-140

**Published:** 2011-05-25

**Authors:** Kyung Mo Kim, Gustavo Caetano-Anollés

**Affiliations:** 1 Evolutionary Bioinformatics Laboratory, Department of Crop Science, University of Illinois, Urbana, IL 61801, USA; 2 Korean Bioinformation Center, Korea Research Institute of Bioscience and Biotechnology, 111 Gwahangno, Yuseong-gu, Daejeon 305-806, Korea

## Abstract

**Background:**

The last universal common ancestor represents the primordial cellular organism from which diversified life was derived. This urancestor accumulated genetic information before the rise of organismal lineages and is considered to be either a simple 'progenote' organism with a rudimentary translational apparatus or a more complex 'cenancestor' with almost all essential biological processes. Recent comparative genomic studies support the latter model and propose that the urancestor was similar to modern organisms in terms of gene content. However, most of these studies were based on molecular sequences, which are fast evolving and of limited value for deep evolutionary explorations.

**Results:**

Here we engage in a phylogenomic study of protein domain structure in the proteomes of 420 free-living fully sequenced organisms. Domains were defined at the highly conserved fold superfamily (FSF) level of structural classification and an iterative phylogenomic approach was used to reconstruct *max_set *and *min_set *FSF repertoires as upper and lower bounds of the urancestral proteome. While the functional make up of the urancestral sets was complex, they represent only 5-11% of the 1,420 FSFs of extant proteomes and their make up and reuse was at least 5 and 3 times smaller than proteomes of free-living organisms, repectively. Trees of proteomes reconstructed directly from FSFs or from molecular functions, which included the *max_set *and *min_set *as articial taxa, showed that urancestors were always placed at their base and rooted the tree of life in Archaea. Finally, a molecular clock of FSFs suggests the *min_set *reflects urancestral genetic make up more reliably and confirms diversified life emerged about 2.9 billion years ago during the start of planet oxygenation.

**Conclusions:**

The minimum urancestral FSF set reveals the urancestor had advanced metabolic capabilities, was especially rich in nucleotide metabolism enzymes, had pathways for the biosynthesis of membrane *sn1,2 *glycerol ester and ether lipids, and had crucial elements of translation, including a primordial ribosome with protein synthesis capabilities. It lacked however fundamental functions, including transcription, processes for extracellular communication, and enzymes for deoxyribonucleotide synthesis. Proteomic history reveals the urancestor is closer to a simple progenote organism but harbors a rather complex set of modern molecular functions.

## Background

Cellular organisms in the contemporary living world have been classified into superkingdoms Archaea, Bacteria, and Eukarya [1] ever since archaebacteria were discovered over three decades ago [2]. Every newly recorded species of the over a million that have been described (belonging to the ~10^7^-10^8 ^that probably exist on Earth [3-5]) has failed to escape from the boundary of the three-superkingdom natural system. This system confers a rigid universal taxonomic structure for a universal 'tree of life', a phylogeny that describes how lineages on Earth diversified from a primordial ancestor. In phylogenetics, the most basal and ancient (plesiomorphic) node of an evolutionary tree defines a common ancestor of the organismal set (taxa) under study. Generally, this node is a hypothetical entity, the first of a chain of ancestors giving rise to each and every organism in the tree. The tree of life defines the last universal common ancestor (LUCA), an organism responsible for the emergence of Earth's primary lineages [6-12]. However, the current tree of life is not universal, i.e. not all primary lineages are represented in the tree. The tree describes the evolution of organisms with ribosome-containing cells (ribocells) and does not incorporate viruses or other lineages that lack ribosomes (virocells), have biological boundaries that are difficult to define, or are evolutionarily highly mobile [13,14]. The tree of life may also have reticulations because of horizontal gene transfer (HGT), convergent evolution, and recruitment processes that complicate the genetic make up of lineages. While the terms "network" or "rhizome" have been proposed, reticulations often affect the history of organismal components but maintain the integrity of lineages [15], and all lineages most probably had a single evolutionary origin [16]. LUCA is believed to be a cellular entity, even though its make up has been considered contentious [6,17]. Its cellular status was probably attained progressively, starting with molecular components drawn from the emerging and fuzzy biochemistry of primordial Earth and ending with more complex biological machinery needed to sustain the integrity of lineages in an increasingly diversified world. In particular, cell-defining ribosomes evolved from the start as ribonucleoprotein ensembles [18] but emerged before the loss of the first protein fold in a superkingdom 2.6 billion years (giga-annum; Ga) ago, a first indication of clear organism diversification [19,20]. Conceptually, the primordial ancestor by definition cannot represent a formal lineage; no prior phylogenetic ancestor precedes it, being first and last of a gradually evolving community of primordial organisms. Consequently, we regard this primordial ancestral entity as the most basal node of the tree of life and name it for simplicity the 'urancestor' (root 'ur' = primitive), a primitive organism that evolved in a time when improving the molecular make up was the main focus of the evolutionary progression.

The emergence of the urancestor probably represents a singular evolutionary chain of events responsible for the universal genetic code and widely shared biological structure. However, defining the complexity (or simplicity) of its molecular features (characters) is problematic, as it often depends on the levels of molecular structure analyzed (e.g., molecular sequence, motifs in sequence, structural motifs, structural domains), the rooting of the tree of life, and the methods used for ancestral character state reconstruction. While it is likely that the urancestor had functional ribosomal RNA (rRNA) and some transfer RNA (tRNA) molecules [6,8,21], as revealed by phylogenetic analysis of RNA and protein structure [18,22]), and was probably endowed with functions associated with DNA replication [23], its translational apparatus has been considered to be rudimentary [6,24]. Penny and Poole [8] later on expanded urancestral complexity to include besides translation and DNA replication, transcription, cell division, and regulatory elements of information processing, suggesting a functionally complex entity much akin to that of modern life. Comparative genomic studies that included the first archaeal genome that was sequenced, *Methanococcus jannaschii*, resulted in arguable conclusions [25]. Broader comparative analyses of entire genomic repertoires were still inconclusive, supporting the simple 'progenote' model proposed by Woese [6] or the complex 'cenancestor' (*sensu *[26]) model that equated the urancestor to modern organisms [7-12]. For example, Koonin [9] traced evolutionary histories of orthologous genes in ~100 genomes using parsimony thinking and phylogenetic trees of ribosomal proteins and rRNA. Although the numbers of urancestral genes ranged from tens to ~1,800 depending on composite parameter values of rates of differential HGT and gene loss, the gene set included most of the translation apparatus and a few transcriptional components but lacked DNA replication regardless of parametric values. In contrast, Ouzounis and colleagues initially identified ~300 ancient proteins from homologues of *M. jannaschii *open reading frames [7]. Except for regulatory elements related to information (i.e. translation, transcription, etc), proteins encompassed Penny and Poole's urancestral functions. A more recent study of 184 genomes identified 669 orthologous protein families, which cover 561 detailed functional classes that are involved in almost all essential biological processes of extant life, including translation, transcription and its regulation, DNA replication, recombination, and repair, transport and membrane-associated functions, electron transfer, and metabolism [10]. Similarly, comparison of protein fold structures among lineages of the three superkingdoms supported an urancestor with functional complexity similar to that of extant life [11,27].

Mutation and chromosomal rearrangement change the sequence of nucleic acids and proteins continuously. The high pace of sequence change can complicate phylogenetic analysis and character state reconstruction. Differential evolutionary rates among organismal lineages, horizontal gene transfer (HGT), and non-orthologous gene displacement can generate phylogenetic artifacts such as long-branch attraction and unrecognized paralogy [8,9,15,28]. This compounds with difficulty in identifying homology by sequence alignment [29], the troublesome task of assigning orthologous relationships [28], and the problem of saturation of substitutions [15]. These phylogenetic artifacts can be responsible for the variable rootings of the tree of life. Finally, origins of genes can be highly variable depending on reference trees that are used for tracing their evolutionary histories. Except for a couple of studies [11,27], all surveys of urancestral molecular repertoires that were fully based on sequence conservation must be considered susceptible to these problems.

In this study we focus on the three-dimensional (3D) structure of protein domains that result from the folding of polypeptide chains. These structures are grouped into fold families (FF), fold superfamilies (FSFs), and folds (F) in a robust hierarchical classification scheme, the structural classification of proteins (SCOP)[30]. In particular, we focus on FSFs, groups of FFs (protein structures that are homologous based on sequence identity) that share structural and functional features suggestive of a common evolutionary origin. SCOP currently defines ~2,000 FSFs, and ~7 × 10^7 ^proteins present in ~1,124 completely sequenced genomes (October 2009)[31] have been assigned to at least one FSF by scanning with hidden Markov models (HMMs)[32]. The relatively small number of FFs, FSFs, and Fs present in nature indicates protein structure is much more conserved than sequence and is refractory to evolutionary change [27]. In fact, a comparative analysis of structurally aligned protein domains and aligned protein sequences showed structures are 3-10 times more conserved than sequences [33]. The structures are therefore good phylogenetic markers for deep events in evolutionary history. The large and growing number of genomic sequences and their associated FSF assignments also guarantees a broad organismal census. Moreover, the evolutionary conservation and deep phylogenetic signal of FSFs has been repeatedly verified by recovery of reliable phyletic patterns describing the evolution of the three superkingdoms [34,35] and by successful exploration of the origin of modern metabolic networks [36]. All of these features and the fact that domains diversify mostly by vertical descent [37-39] make domain structure extremely useful for the evolutionary study of proteomes and urancestral protein repertoires.

**Figure 1:**
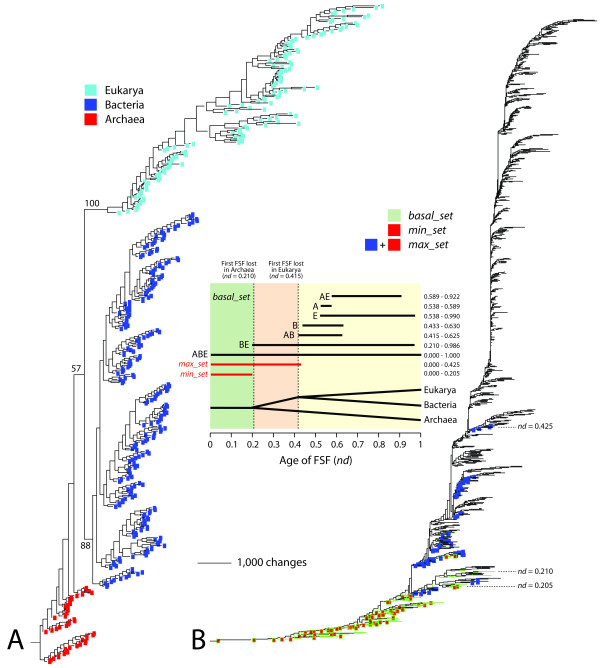
**Phylogenomic trees of proteomes and domain structures**. A. Phylogenomic tree of proteomes describing the evolution of 420 FL organisms. One most parsimonious tree was recovered from an analysis of the abundances of 1,420 FSFs in the proteomes of the FL organisms (1,388 parsimoniously informative sites; 130,844 steps; CI = 0.075; RI = 0.774; *g*_1 _= -0.199). Taxa are proteomes and characters are FSFs. Non-parametric bootstrap values that have more than 50% supports were shown above or below branches that cluster the superkingdoms or much higher groups. Terminal leaves of Archaea, Bacteria, and Eukarya were labeled in red, blue, and cyan, respectively. B. A phylogenomic tree of protein domain structure describing the evolution of 1,420 FSFs. One most parsimonious tree was recovered from analysis of genomic abundances of FSFs in 420 FL proteomes (420 parsimoniously informative sites; 201,838 steps; CI = 0.046; RI = 0.806; *g*_1 _= -0.0187). Taxa are FSFs and characters are proteomes. Terminal leaves were not labeled, since they would not be legible. INSET: A timeline was unfolded from the tree of FSFs using a PERL script that calculates the relative age (node distance; *nd*) of individual FSFs by counting the number of internal nodes along a lineage from the root to a terminal node on a relative 0-1 scale. The bar diagram shows ranges of age (*nd*) for FSFs that are unique to superkingdoms (A, B, or E) or are shared by two (AB, AE, or BE) or all (ABE) superkingdoms. The basal_set represents the set of FSFs that appeared before the first appearance of an FSF not shared by all superkingdoms, a BE FSF.

Here we build a tree of life using a census of FSF domains in 420 free-living (FL) proteomes and employ an iterative strategy to reconstruct the urancestral proteome (Figure 1). Initially, FSFs that are placed in the root branch of the tree are assigned to the urancestor. We reason that FSFs that appear after the division of the three superkingdoms must be absent in a non-diversified organismal world. We therefore exclude FSFs of relatively recent age iteratively, using FSFs in the root branch of an initial proteome tree as phylogenetic characters for reconstructing a new tree. The procedure identifies a set of urancestral FSFs that is shielded from the so-called 'modern effect', the impact of recent convergent evolutionary processes on ancient repertoires [40]. Finally, biological functions associated with upper and lower bounds of an urancestral proteome are annotated using a coarse-grained functional classification of FSFs [41]. This information served to define the molecular functions and biological processes of the primordial ancestor of diversified life and allowed to time the actual appearance of this elusive but crucial ancestral entity.

## Results

### Genomic census and reconstruction of a Tree of Life

In this study we conducted a genomic census of protein domains at FSF level of structural complexity in organisms that have been completely sequenced and used this information to build trees of life describing the evolution of proteomes. We first analyzed 645 complete proteomes in 49 archaeal, 421 bacterial, and 175 eukaryotic species (the Total set). Using an *E*-value cutoff of 10^-4^, we extracted reliable proteomic HMM hits for 1,531 FSFs, 1,446 of which covered ca. 80% of the total FSFs defined in SCOP. We then generated intrinsically rooted phylogenomic trees directly from FSF abundances in the 645 proteomes. A most parsimonious reconstruction of a tree of life (Total-tree) showed that organisms in the three superkingdoms formed distinct groups (Additional file 1, Figure S1). Eukaryotic proteomes were placed in a monophyletic group that was supported by a 50% bootstrap support (BS) value. In turn, archaeal and bacterial proteomes were para- and polyphyletically grouped, respectively. A manual analysis of organismal lifestyles using various sources of information revealed that proteomes from obligate and non-obligate parasitic organisms (225 in total) were distributed throughout the tree but generally occupied the most ancient branches of the superkingdom groups (e.g. *Mycoplasma *spp. and *Rickettsia *spp. in Bacteria; *Cryptosporidium *spp. and *Plasmodium *spp. in Eukarya). Basal organisms included notable obligate parasites (*N. equitans *in Archaea, *Chlorobium chlorochromatii *in Bacteria, and *Encephalitozoon cuniculi *in Eukarya).

The genomes of organisms that are parasitic or that establish symbiotic relationships with other organisms have frequently experienced reductive evolution, discarding enzymatic and cellular machineries in exchange for resources from their hosts. Since the inclusion of these genomes can lead to incorrect phylogenetic trees [15,42], we excluded genomes from all but 420 FL organisms. This FL set included 48 archaeal, 239 bacterial, and 133 eukaryotic organisms. The exclusion of non-FL genomes resulted in 26 FSFs that were absent in the proteomes of FL organisms. We therefore reconstructed a most parsimonious rooted tree that described the evolution of FL organisms from genomic abundances of the remaining 1,420 FSF. The tree of FL proteomes (FL-tree) also supported the trichotomy of the superkingdoms (Figure 1A). Unlike the Total-tree, the FL-tree showed that archaeal species formed a polyphyletic group and were basal in the tree. The proteomes of Bacteria and Eukarya formed strong monophyletic groups supported by 88 and 100% BS values, respectively. A sister-clade relationship between Bacteria and Eukarya existed but was poorly supported (57% BS). While statistics describing the skewness [43] of the Total-tree were close to zero (*g*_1 _= -0.065), the FL-tree had more significant phylogenetic structure (*g*_1 _= -0.199, *P *< 0.01). Our focus therefore centered on FL-tree reconstructions.

In the FL-tree, the number of bacterial proteomes dominates those of organisms in other superkingdoms. It is thus possible to argue that the bias of sampled proteomes per superkingdom can cause long-branch attraction, which can eventually lead to incorrect deep phylogenetic relationships. We thus sampled equal numbers of proteomes per superkingdom and replicated trees of proteomes. We first chose only one proteome per genus in Archaea because the numbers of proteomes vary depending on individual archaeal genera. As a result, we selected 34 out of 48 archaeal proteomes. We then classified bacterial and eukaryal proteomes at the phylum level of the two superkingdoms. While some phyla contained large number of proteomes (e.g., 50 proteomes in Firmicutes), some other contained very few (e.g., only two proteomes in Acidobacteria). We thus randomly sampled bacterial and eukaryal proteomes whose numbers are proportional to the size of corresponding phyla and then adjusted the number of sampled proteomes per superkingdom to the sample size of Archaea (34 proteomes). By following the same steps described in Methods, the genomic abundances of 1,370 FSFs in the balanced set of 102 proteomes resulted in one most parsimonious tree (Additional file 1, Figure S2). The phylogenetic relationships among the three superkingdoms that we found in the FL-tree of 420 proteomes ([Archaea, [Bacteria, Eukarya]]; Figure 1A) were consistently present in the newly reconstructed tree (Additional file 1, Figure S2). Furthermore, monophyly of Bacteria and Eukarya, and paraphyly of Archaea were maintained. These results indicate our phylogenetic approach based on genomic abundance is robust against uneven sampling of proteomes in superkingdoms.

### Iterative refinement to identify urancestral FSFs

**Figure 2:**
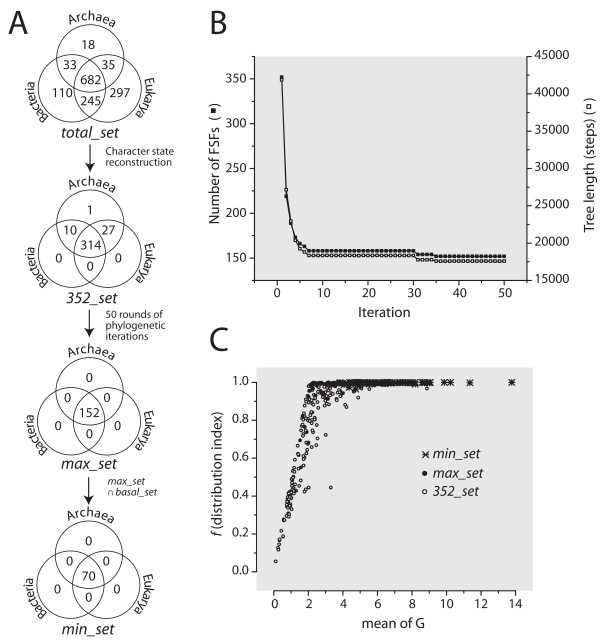
**Defining urancestral FSFs by serial and iterative phylogenetic and character state reconstruction analysis**. A. Venn diagrams with seven cells describing the distribution of FSFs in the three superkingdoms of life, Archaea (A), Bacteria (B), and Eukarya (E), in sets derived from iterative refinement. The Venn diagram in the top shows the taxonomic occurrence of the modern set of 1,420 FSFs in the 420 FL organisms analyzed in this study. The Venn diagram of the *352_set *describes the distribution of 352 FSFs that are plesiomorphic and positioned at the root branch of the proteome tree of Figure 1A. The Venn diagram of the urancestral *max_set *describes the distribution of a minimal set of 152 FSFs that was recovered by serial and iterative phylogenetic and character state reconstruction analysis (*see *B). The set was defined by identifying a tree of proteomes with minimal length, extracting FSF that are plesiomorphic and basal, and updating the character set of FSFs for further phylogenetic analysis in 30 chains of 50 rounds of iterations each. The Venn diagram of the *min_set *describes the distribution of 70 FSFs identified by the intersection of the *max_set *and a *basal_set *of 130 FSFs that are present in the three superkingdoms and are placed at the base of the tree of domain structure described in Figure 1B. B. Change in the length of trees and number of FSFs recovered in individual iterations of chain number 26. For the *i*^*th *^iteration, the left y-axis indicates the number of distinct FSFs that appear in the root branch of the *(i-1)*^*th *^proteome tree and the right y-axis represents the lengths of the reconstructed tree. The numbers of FSFs converged into a single value after the 35^th ^iteration. The arrow points to the iteration that generates the most parsimonious trees with the minimal tree length in the chain. C. Correlation between mean of *G *and *f *indexes for the different sets of FSFs.

We traced gains and losses (character state changes) of characters (FSFs) along the branches of the FL-tree using the APOLIST option in PAUP* [44]. A set of 352 plesiomorphic FSF characters (the *352_set*) was positioned at the root branch. Most FSFs in the *352_set *were present in the three superkingdoms (Figure 2A). These ancient FSFs exhibited only gains (no losses were detected) and were assigned as initial set of characters to each of 30 different iterative chains to define the set of urancestral FSFs. In individual chains, FSFs that are positioned at the root branch of the *X*^*th *^proteome tree were used as character set for building the *(X+1)*^*th *^proteome tree, where the iteration number *X *ranges from 0 to 49. All of the proteome trees that were generated during the iterations showed absence of loss of FSFs in their root branches. The numbers of distinct FSFs and tree lengths decreased dramatically in early iterations of all the chains, especially before iteration 10, and converged to values with little variance (Figure 2B). Some of the iterations produced two or more equally parsimonious trees. The numbers of plesiomorphic FSFs and tree lengths for over 1,500 most parsimonious trees that were retained ranged from 152 to 219 and from 17,576 to 18,215 steps, respectively. The minimal set of 152 FSFs was observed in 107 iterations of four different chains (e.g., iterations 35 to 50 of chain 26; Figure 2B). However, one most parsimonious tree (iteration 36 of chain 26) belonged to the set of trees that generated the smallest number of the FSFs (152; Figure 2B).

In general, some of character-state changes on a given phylogenetic tree can be ambiguous. That is, these changes can occur on different branches without increase or decrease in the number of steps of the tree. In the root branch of the FL-tree, 50 out of the 352 FSFs had character-state changes that were ambiguously assigned. However, the ambiguity sharply decreased in the early iterations of every chain as shown in Additional file 1, Figure S3. Consequently, all of the 152 FSFs that were obtained from iteration 36 onwards of chain 26 had purely unambiguous character-state changes.

To remove FSFs that might be derived from ancient HGT or convergent evolutionary processes, we used information in a rooted tree of domain structure that describes the evolution of 1,420 FSFs (taxa) and was reconstructed from FSF abundance in the proteomes of 420 FL organisms (characters). In this tree, the most basal 130 FSFs were present in the three superkingdoms and appeared before the rise of the first FSF that was completely lost in a superkingdom (Archaea) (Figure 1B). The intersection of this ancient set of 130 FSFs with the set of 152 FSFs defined 70 urancestral FSFs. Iteration analysis of this new urancestral FSF set with the same numbers of chains and iterations showed there was no further decrease in the number of FSFs, although tree lengths varied slightly from 10,271 to 10,284 steps. We therefore define the sets of 152 and 70 urancestral FSFs as the maximum (*max_set*) and minimum (*min_set*) boundaries of the urancestral FSF repertoire, respectively. The biological rationale for the use of the iterative analysis and the tree of domain structures can be found in Discussion.

### Taxonomic distribution of urancestral FSFs

Under the 3-superkingdom taxonomic system, an individual FSF can be unique to superkingdom Archaea (A), Bacteria (B) or Eukarya (E), or can be shared by two (AB, BE, or AE) or all three superkingdoms (ABE). The initial *352_set *of FSFs that were basal and plesiomorphic consisted of 314 ABE, 10 AB, 27 AE, and 1 A FSF (Figure 2A). As expected, the *max_set *of 152 urancestral FSFs (and the *min_set *of 70 FSFs embedded in it) had only ABE domains. To describe the popularity of urancestral FSFs in proteomes, we adopted two different indexes: (1) a distribution index *f*, the fraction of organisms that contain a certain FSF (in a 0-1 scale); and (2) the mean of *G*, the sum of *g *values of all sampled proteomes for a particular FSF divided by the total number of proteomes. The *352_set *had *f *and mean of *G *values that ranged 0.055-1 and 0.114-13.781, respectively (Figure 2C). The *max_set *has corresponding values that ranged 0.694-1 and 1.529-13.781 and the *min_set *had values that ranged 0.964-1 and 3.807-13.781. Clearly, *min_set *FSFs are ubiquitous and highly represented in proteomes.

### FSF architectures and functions of minimum and maximum urancestral sets

**Figure 3:**
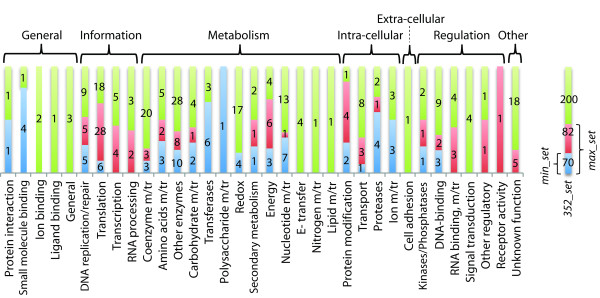
**Distribution of molecular functions in the FSF *352_set *that encompasses the urancestral *max_set *and *min_set***. In the diagram, the *min_set *has 70 FSFs. A total of 82 FSFs are unique to the *max_set *when compared to the *min_set*. The 200 remaining FSFs are not included in both of the urancestral sets. The bar graph displays 49 coarse-grained functional sub-categories (bottom labels) in seven major categories (top labels) defined by Vogel and Chothia [41]. Bars describe the number of FSFs in different sets, with heights normalized for sub-categories. The bars in the diagram show the initial appearance of molecular functions in the *min_set *or their additional accumulation in the *max_set *or *352_set *for individual sub-categories. For example, the sub-category *translation *in *Information *has 6, 28, and 18 FSFs, that are included in the *min_set*, the 82 FSFs of the *max_set*, and the 200 FSFs of the *352_set*, respectively. Here, 6 FSFs are common in all of the three sets, 28 FSFs are absent in the *min_set *but appear in the *max_set *and *352_set*, and 18 FSFs are unique to the *352_set*.

For each FSF that belongs to the *352_set*, the *max_set*, and the *min_set*, we tabulated molecular functions defined by SCOP coarse-grained functional classification, mean of *G *and *f *indices, and superkingdoms distribution in Additional file 1, Table S1. Since it is difficult to describe the urancestor based on FSF structural and functional information, we grouped urancestral FSFs within seven major categories and 49 sub-categories of functions. To avoid confusion, category names are displayed in italics and the initial letters are capitalized (*General*, *Information*, *Metabolism*, *Intra-cellular processes*, *Extra-cellular processes*, *Regulation*, and *Other*). While all seven major categories were present in the *352_set*, the *max_set *of urancestral FSFs involved six (all except for *Extra-cellular processes*) and the *min_set *involved only five (all except *Extra-cellular processes *and *Other*) (Figure 3). The FSFs of the three sets were distributed in 34, 26, and 20 sub-categories, respectively (Figure 3; Additional file 1, Table S2). For *General*, both the *max_set *and the *min_set *had five FSFs that belong to sub-categories *protein interaction *and *small molecule binding *while the *352_set *had 13 FSFs with additional functions related to binding activities (i.e. ion and ligand binding; Figure 3). For *Information*, the profile of sub-categories for the *max_set *was identical to that for the *352_set*, although the former set had fewer FSFs than the latter for individual sub-categories (Figure 3). The functions of the *max_set *encompassed *DNA replication/repair*, *translation*, *transcription*, and *RNA processing*, where the last two categories were absent in the *min_set*. In the three sets, the *nuclear structure *and *chromatin structure *functions essential for developing the eukaryotic cell were absent. For *Metabolism*, the FSFs of the three sets were commonly distributed in *coenzyme m/tr*, *amino acids m/tr*, *other enzymes*, *carbohydrate m/tr*, *transferases*, *polysaccharide m/tr*, *redox*, *secondary metabolism*, *energy*, and *storage*, where 'm/tr' means metabolism and transport (Figure 3; Additional file 1, Table S2). FSFs that function in *photosynthesis *and *cell envelope m/tr *were completely absent in all of the sets. While few FSFs of *electron transfer*, *nitrogen m/tr*, and *lipid m/tr *were present in the *352_set*, they were absent in both the urancestral *max_set *and *min_set*. For *Intra-cellular processes*, subcategories *protein modification*, *transport*, *proteases*, and *ion m/tr *were shown by all of the sets while *cell cycle related to apoptosis*, *phospholipid m/tr*, *cell motility*, and *trafficking/secretion *were absent in all. Only one FSF of the *Extra-cellular processes *category was present in the *352_set *and belonged to sub-category *cell adhesion *(out of four in the category). For *Regulation*, *min_set *FSFs were *kinases/phosphatases *and *DNA-binding *and *max_set *FSFs were additionally involved in *receptor activity *and *other regulatory functions*. The sub-category *Signal transduction *was present in the *352_set*, but not in the *max_set *and the *min_set*. Five and 23 FSFs that are grouped into *unknown function *of category *Other *were assigned to the *max_set *and the *352_set*, respectively. Two FSFs in the *352_set *were not annotated in sub-categories and are labeled NONA in Additional file 1, Table S1.

### Comparing the functions of urancestral FSF sets and extant proteomes

**Figure 4:**
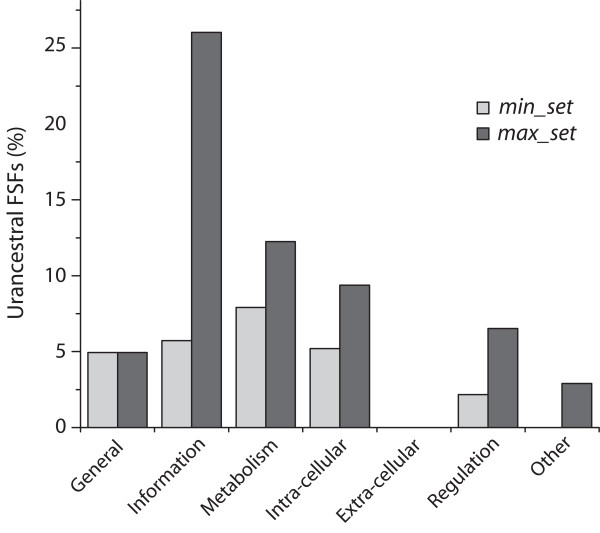
**The general molecular functions of urancestral FSF sets relative to those present in the modern protein world**. Bars describe the relative proportion of FSFs that are prent in urancestral sets and are annotated to the corresponding major functional categories were displayed on the top of the bars. For each of the major categories, the height of a bar was calculated by dividing the number of FSFs in urancestral sets by the number of FSFs that are in the global set of 1,416 FSFs that are present in the 420 FL proteomes examined. Note that 4 FSFs were not assigned to any of the categories.

The functional repertoire of urancestral FSFs was compared to that of the set of 1,420 FSFs that are present in the 420 extant FL proteomes we analyzed. Since 4 FSFs were not annotated, the remaining 1,416 FSFs were used as a the reference (extant) set and included 101 FSFs in *General*, 192 in *Information*, 506 in *Metabolism*, 192 in *Intra-cellular processes*, 69 in *Extra-cellular processes*, 184 in *Regulation*, and 172 in *Other*. Less than 10% of these FSFs were present in the urancestral *min_set *(Figure 4). With the exception of FSFs linked to *Information *(with more than 25% of extant FSFs), less than 15% of extant FSFs in the remaining categories were present in the urancestral *max_set*. Within the *min_set *and *max_set*, *Metabolism *was the most abundant (i.e. 40 out of 70 FSFs in the *min_set*; 62 out of 152 FSFs in the *max_set*). However, while the most abundant category of the *min_set *was *Metabolism*, *Information *was prevalent in the *max_set *(Figure 4). To examine which sub-functional categories were enriched in the urancestral sets, we conducted a statistical test for each of them based on the hypergeometric distribution (for a complete list, see Additional file 1, Table S3). The analysis revealed that three and two categories were enriched in the *min_set *and the *max_set *with more than 95% confidence level, respectively (Table 1). The enrichment of *nucleotide m/tr *of the category *Metabolism *was supported by both of the urancestral sets. In turn, *small molecule binding *of *General *and *transferases *of *Metabolism *were uniquely enriched in the *min_set*, and *translation *of *Information *was enriched only by the *max_set*.

**Table 1:** The enrichment of molecular functions in the urancestral FSF sets

Urancestor	Functional sub-categories (categories)	Urancestral FSFs (sample)	Extant FSFs (bkgd)	Rate for sample	Rate for bkgd	Ratio	*P *value
*min_set*	*Transferases (Metabolism)*	6	29	0.066	0.020	4.19	0.0021
	*Small molecule binding (General)*	4	22	0.057	0.016	3.68	0.021
	*Nucleotide m/tr (Metabolism)*	7	29	0.100	0.020	4.88	0.00037
*max_set*	*Translation (Information)*	34	89	0.224	0.063	3.56	E^-12^
	*Nucleotide m/tr (Metabolism)*	8	29	0.053	0.020	2.57	0.0083

We evaluated which of 49 sub-categories are enriched by the *min_set *or the *max_set*. In the table, *sample *means the *min_set *(70 FSFs) or *max_set *(152 FSFs). The 1,416 FSFs represent the *background *(bkgd). For convenience, let us assume that *M *and *k *represent the number of FSFs that are assigned to a particular sub-category in the *background *(extant FSFs) and *sample *(urncestral FSFs) and *N *and *n *are the total numbers of FSFs in the *background *and *sample *without functional categorizations. The rates for the *sample *and the *background *are notated by *k/n *and *M/N*, respectively. The ratio was determined by dividing the sample rate by the background rate. The *P *values were calculated using the hypergeometric distribution. The enrichment of the sub-categories that are listed was supported at the 95% confidence level.

### Structural complexity of the urancestor

**Figure 5:**
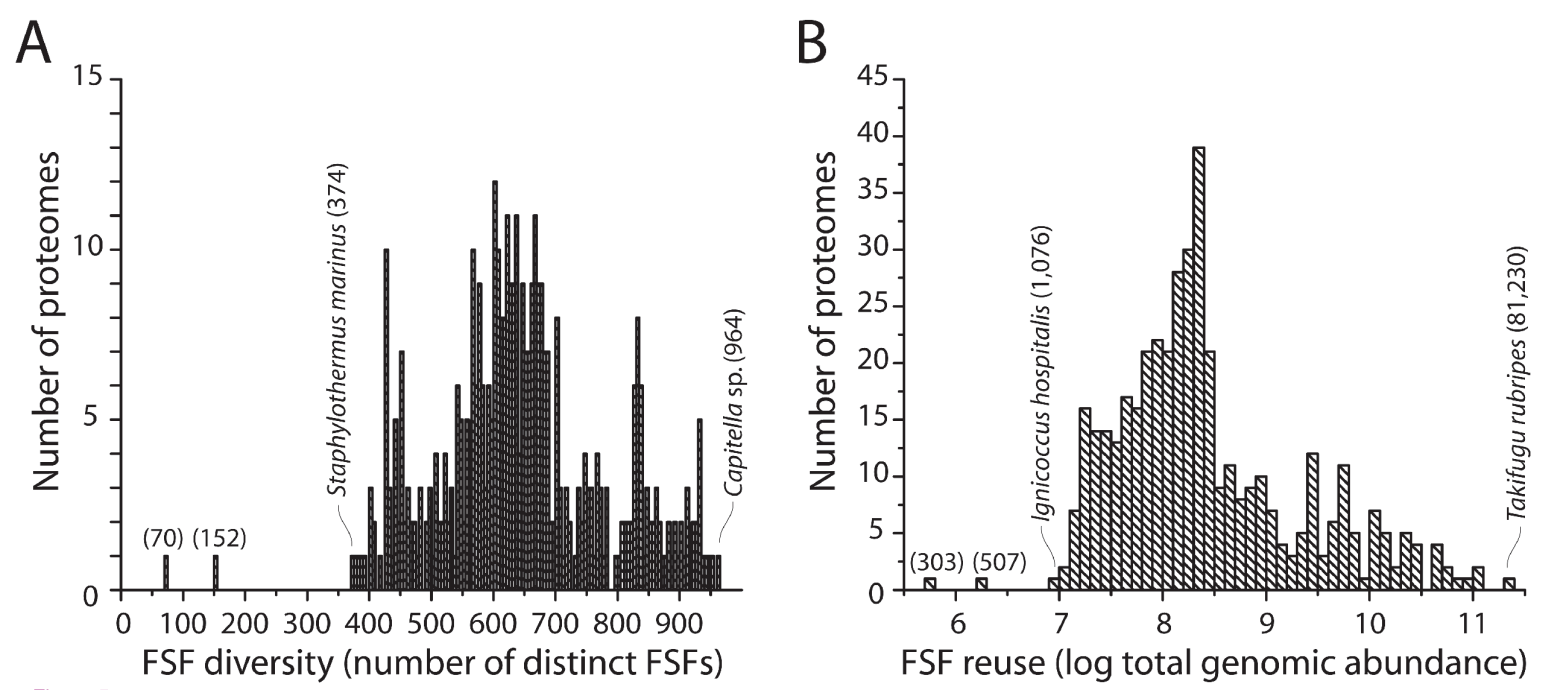
**Diversity and reuse of FSFs in proteomes**. A. Frequency distribution plot describing the number of proteomes for increasing numbers of distinct FSFs in individual proteomes, in bins of 5. The FSF numbers of the *min_set*, *max_set*, and the smallest and the largest proteomes are given in parentheses. B. Frequency distribution plot describing the number of proteomes for increasing levels of FSFs reuse in individual proteomes. Reuse scales multiple occurrences of FSFs in proteomes and is logarithmically normalized.

In order to determine if urancestral FSF sets were simpler in number than FSFs of extant organisms, we checked the number of distinct FSFs (FSF diversity) for each of the 420 FL proteomes sampled (Figure 5A). The numbers of distinct FSF ranged from 374 in *Staphylothermus marinus *(a sulfur-reducing hyperthermophilic archaeon) to 964 FSFs in *Capitella *sp. (a polychaete worm). Proteomic occurrence levels of extant FSFs showed that over 200 additional FSFs are necessary in urancestral FSF sets to account for the complexity of the simplest organism in existence today.

We also compared the abundance of urancestral FSFs (FSF reuse) to that of extant organisms (Figure 5B). While it is difficult to infer multiple appearances of individual FSFs in organisms before the advent of the three superkingdoms, proteome trees were generated from FSF abundance levels (*g *values) and provide histories of character state changes for individual characters (FSFs). We therefore counted how many gains or losses of individual urancestral FSFs occurred in the root branches of the trees. In the process of identifying the two urancestral FSF sets, we obtained most parsimonious trees of proteomes in iterations that generated the 70 and 152 FSF sets. In these trees, *min_set *and *max_set *FSFs appeared 303 and 507 times without any event of loss, respectively. Using a similar rationale, we counted gains and losses of FSFs in individual proteomic lineages by traveling from the root to terminal leaves of the proteome tree. Total abundance levels for FSFs in each of the 420 FL proteomes (i.e. the total number of FSFs in a proteome) ranged from 1,076 to 81,230. The proteome that had the lowest total abundance level was *Ignicoccus hospitalis*, a chemolithoautotrophic and hyperthermophilic archaeon (Figure 5B). As expected, the FSF abundance levels of the urancestral sets were simpler than those of any FL proteome in existence today.

**Figure 6:**
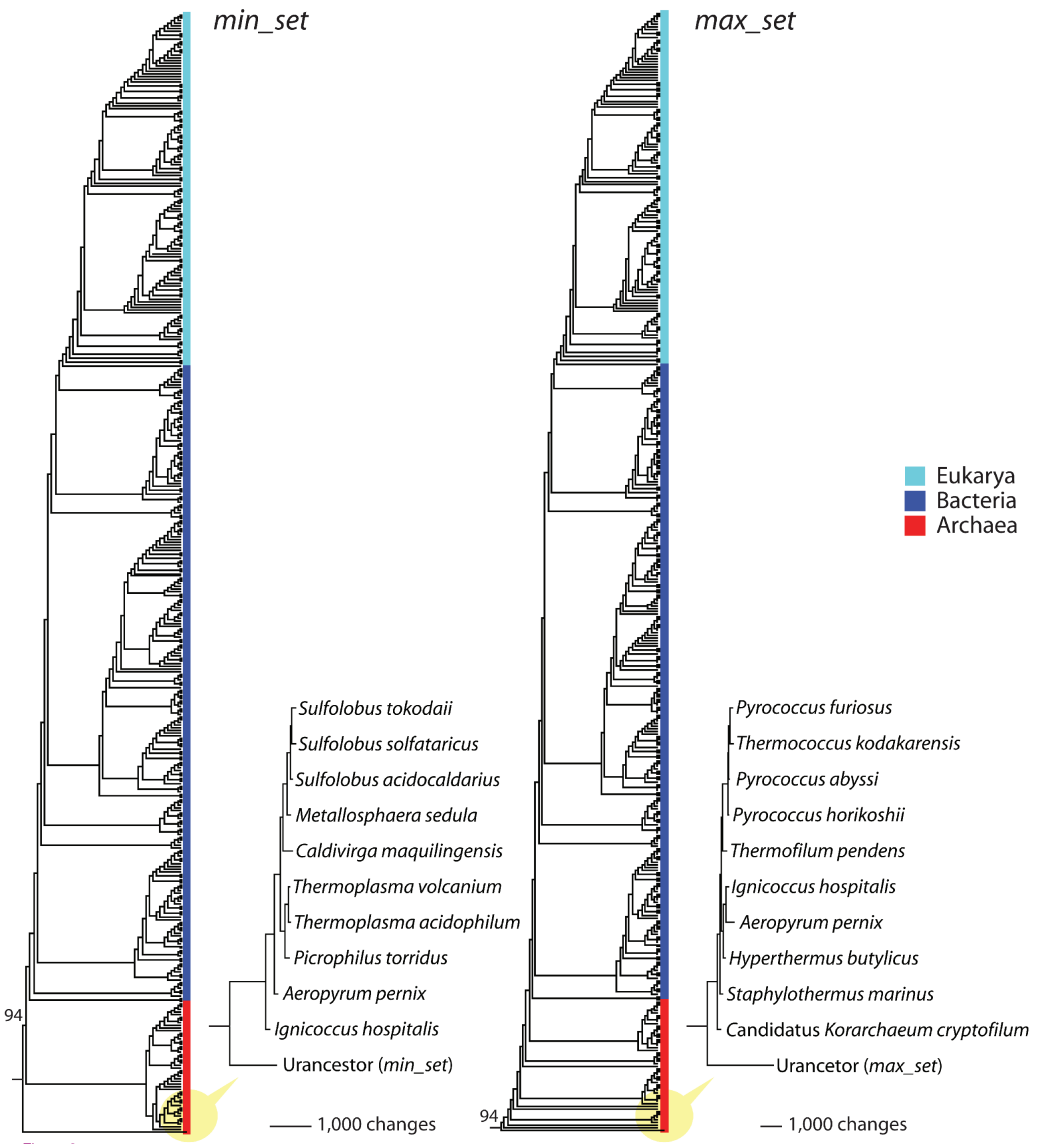
**Phylogenetic placement of the urancestor in trees of proteomes reconstructed from FSF domain structure**. A single most parsimonious tree of proteomes that included the urancestral *min_set *(1,388 parsimoniously informative sites; 132,829 steps; CI = 0.080; RI = 0.773; *g*_1 _= -0.3354) or the *max_set *(1,388 parsimoniously informative sites; 134,801 steps; CI = 0.090; RI = 0.773; *g*_1 _= -0.239) as artificial taxa was recovered from a maximum parsimony analysis of genomic abundance levels of 1,420 FSFs (characters) in 421 proteome (taxa). Cladograms and subtrees containing the urancestor and a set of 10 taxa that were the most basal in the trees show that urancestral sets are more ancient than any of the 420 FL proteomes that were analyzed. Phylogenetic relationships with BS > 50% are displayed above branches.

To strengthen the quantitative results presented above, we reconstructed trees of proteomes that included the urancestral proteomes as taxa. These trees illustrate the evolution of the 420 FL organisms and that of the urancestor if this entity existed today. We defined the two urancestral FSF sets as artificial proteomes and assigned character states (N) to abundance levels of urancestral FSFs. Because character states represent maximum transformed *g *values, the urancestor had maximum abundance levels for the given urancestral FSFs. On the other hand, we assigned '0' to the FSFs that were not present in the urancestral sets. Each of the urancestral proteomes was separately added to the matrix of 1,420 FSFs and 420 FL proteomes, and proteome trees were reconstructed that included the *min_set *and *max_set *urancestor (Figure 6A and 6B). In both cases, the artificial urancestral proteomes (resurrected in silico) appeared at the base of the trees supported by 94% BS levels, and were basal to the most basal archaeal proteomes in the tree. Furthermore, over 2,000 character-state changes explicitly discriminate the artificial urancestors from other FL proteomes as shown in the sub-trees that displays the 10 most ancient taxa (Figure 6).

### Functional complexity of the urancestor

**Figure 7:**
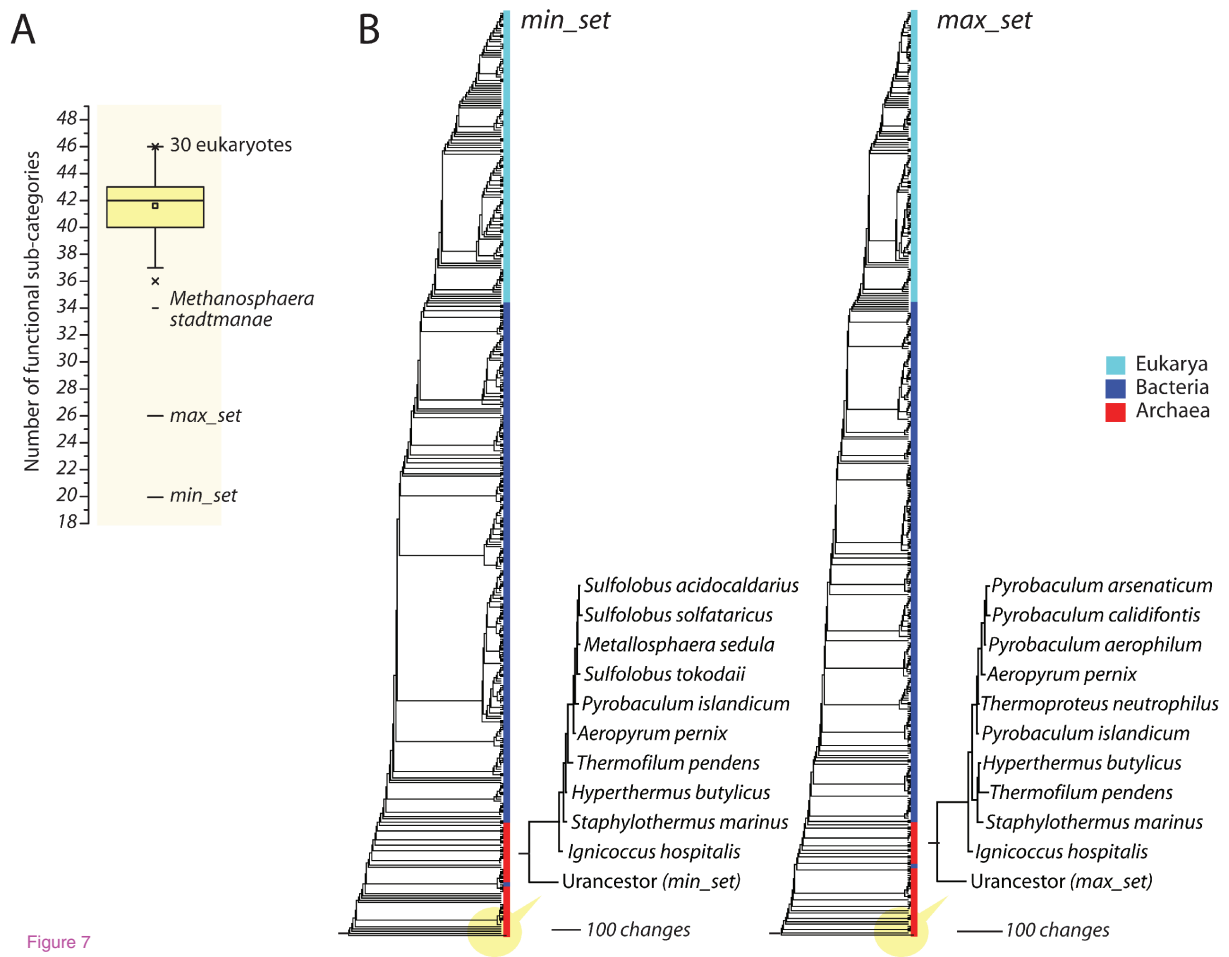
**Phylogenetic placement of the urancestor in trees of proteomes reconstructed from molecular functions**. A. Box plot describing the distribution of the numbers of functional sub-categories (maximum is 49) that are associated with FSF repertoires in urancestral *min_set *and *max_set *and the 420 FL proteomes analyzed. The box was delimited by lower, median, and upper quartiles. Whiskers encompass 5% to 95% values, and crosses 1% and 99% percentiles. Lines on the top and bottom of the plot indicate maximum and minimum numbers. Organisms associated with these values are labeled. The mean is indicated with a rectangle. B. Trees of proteomes and urancestors generated directly from the number of individual functional subcategories. A single most parsimonious tree of proteomes that included the urancestral *min_set *(46 parsimoniously informative sites; 5,269 steps; CI = 0.121; RI = 0.815; *g*_1 _= -0.069) or the *max_set *(46 parsimoniously informative sites; 5,270 steps; CI = 0.121; RI = 0.814; *g*_1 _= -0.133) as artificial taxa was recovered from a maximum parsimony analysis of diversity of molecular functions in proteomes. The matrix contained 421 taxa (i.e. 420 FL proteomes and the urancestral set) and 49 functional sub-categories as characters, with character states describing numbers of functional sub-categories. These trees were identical to each other. Cladograms and subtrees containing the urancestor and a set of 10 taxa that were the most basal in the trees show that urancestral sets are more ancient than any of the 420 FL proteomes that were analyzed. Phylogenetic relationships with BS > 50% are displayed above branches.

In order to determine if the functions of the urancestor were simpler than of extant proteomes, we extracted FSFs from each of the 420 FL proteomes examined and assigned coarse-grained functional sub-categories to them. Their number ranged from 34 to 46 (Figure 7A). The simplest functional profile was that of the simplest methanogenic archaeon, *Methanosphaera stadtmanae*, and the most complex functional profile was exhibited by a set of 30 eukaryotic proteomes. As already described, the functions of FSFs in the *min_set *and the *max_set *were classified into 20 and 26 distinct functional categories, respectively. Consequently, the upper bound urancestral FSF set lacked 8 functional sub-categories when compared to the functions of the simplest FL organism.

Using a strategy similar to the one described above for domain structure, we again regarded the two urancestral FSF sets as taxa and evaluated their phylogenomic positions on the proteome trees. Inspired by a recent phylogenomic analysis of molecular functions that was derived directly from ontological data [45], we defined the 49 functional sub-categories as phylogenetic characters and calculated individual *g *values for FSFs pooled by function. Characters in this analysis have character states that describe the number of distinct FSFs that are assigned to a particular pair of a functional sub-category and proteome. As expected, phylogenomic trees reconstructed from transformed g values for FL proteomes and artificial urancestors showed again that the urancetor was basal in the most parsimonious trees (Figure 7B). While the basal placements of the functional urancestral sets were weakly supported (BS < 50%), over 100 character-state changes between the urancestor and the set of the 10 most ancient taxa (all archaeal in origin) supported the functional simplicity of the urancestor of life.

### Ribosome evolution in the urancestor

Crucial ribosomal proteins act as landmarks of ribosomal evolution [19]. Four FSFs present in universal ribosomal proteins, the nucleic acid-binding protein (b.40.4), ribosomal protein S5 domain 2-like (d.14.1), translation protein (b.43.3), and translation protein SH3-like domain (b.34.5) FSFs, were recruited into ribosomal function prior to a major transition in ribosomal evolution (*nd*_FSF _= 0.173) that is claimed to be responsible for modern protein synthesis [18]. All of these FSFs belong exclusively to the urancestral *min_set *(Table 2). A set of 6 additional FSFs that were found to associate with the ribosome immediately after this transition but before the appearance of L7/L12 protein complex (*nd*_FSF _= 0.329), and ensemble that stimulates the activity of elongation factor G, are all exclusively included in the urancestral *max_set *(Table 2). Primordial protein synthesis was therefore developed in the urancestral lineage.

**Table 2:** Ribosomal proteins used as markers of ribosomal evolution in the urancestor

SCOP ID	FSF	r-proteins	SCOP fold superfamily	*min_set*	*max_set*
50249	b.40.4	S12, S17	Nucleic acid-binding proteins	*+*	*+*
54211	d.14.1	S9	Ribosomal protein S5 domain 2-like	*+*	*+*
50447	b.43.3	L3	Translation proteins	*+*	*+*
50104	b.34.5	L2, L24	Translation proteins SH3-like domain	*+*	*+*
55174	d.66.1	S4	Alpha-L RNA-binding motif	-	*+*
46946	a.156.1	S13	S13-like H2TH domain	-	*+*
56053	d.141.1	L6	Ribosomal protein L6	-	*+*
57716	g.39.1	S14	Glucocorticoid receptor-like (DNA-binding domain)	-	*+*
53137	c.55.4	L18, S11	Translational machinery components	-	*+*
54768	d.50.1	S5	dsRNA-binding domain-like	-	*+*
48300	a.108.1	L7, L12	Ribosomal protein L7/12, oligomerisation (N-terminal) domain	-	*+*

### Placing the appearance of the urancestor in the geological record

**Figure 8:**
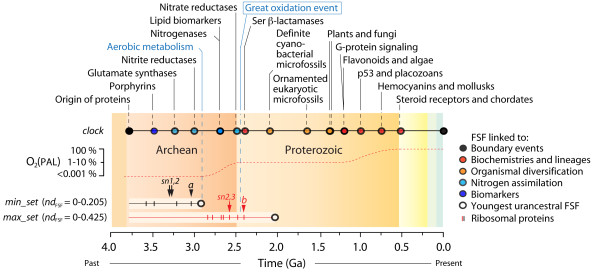
**The rise of the urancestor**. A geological timeline defined by a molecular clock of domain structure at FSF level is used to date the FSF repertoires of the urancestral sets. Oxygen levels are indicated as percentage of present day atmospheric levels (PAL) [70]. Colored circles indicate FSF used for clock calibration. Black and red arrowheads labeled *a *and *b *indicate major and second transitions in ribosomal evolution, respectively [18], and lines indicate the appearance of FSFs associated with ribosomal proteins (table 2). Arrows show the discovery of crucial FSFs linked to membrane glycerol ester and ether lipid chemistries and *sn1,2 *and *sn2,3 *lineages. Time is given in billions of years (Ga).

We used a molecular clock of domain structures based on the phylogenetic tree of FSFs described in Figure 1B to date the appearance of the youngest domain structures in the urancestral *min_set *and *max_set *(Figure 8). The clock was calibrated using FSFs linked to geological ages derived from fossils, events of organismal diversification, and geochemical, biochemical and biomarker data [20]. Plotting fold age (*nd*_FSF_) against geological time (in Ga) revealed a significant linear correlation (*y *= -0.250 *x *+ 0.903; *R*^*2 *^= 0.922, *P *< 0.0001; χ^2 ^= 0.358), which was then used to assign geological age to urancestral FSFs. The age (*nd*_FSF_) of the *min_set *FSFs ranged 0-0.205 and the age of the *max_set *FSFs ranged 0-0.425. The earliest start of organismal diversification therefore occurred sometime between 2.91 and 2.03 Ga ago. Interestingly, the first and second transitions in the evolution of the ribosome occurred before the youngest age of the *min_set *and *max_set *FSFs, respectively (Figure 8), suggesting efficient ribosomal protein synthesis was pre-requisite for organismal diversification.

## Discussion

### A novel phylogenomic strategy identifies the proteome of the urancestor

A number of recent studies have focused on the complexity of the urancestor, its primordial functions, alternative rootings of the tree of life, and the rampancy of HGT [6-12,23,46]. However, results are not congruent, the rooting of the tree of life is still controversial, and the make up of the urancestor remains shrouded in mystery. It is indeed difficult to characterize an entity that existed billions of years ago using information in molecules of life that are modern. When using for example nucleic acid or protein sequences, the pervasive effects of mutation can cloud (saturate) any significant evolutionary signal. Some early studies focused on genomic sequence conservation but suffered for example from the effects of genetic losses and take-overs (e.g., non-orthologous gene displacement [9-11]). The analysis we here report is based on genomic content of protein domain structures at FSF level of the structural hierarchy. FSFs are much more conserved than protein sequence and are highly shared by organismal lineages. About half of FSFs (682 out of 1,420) in the 420 FL proteomes we analyzed in this study are common to the three superkingdoms (Venn diagram; Figure 2A). It is clear that FSFs are more robust against genetic losses and take-overs than corresponding sequences and carry deep evolutionary signatures [36,42,47,48]. Furthermore, the distribution of FSFs in proteomes meets crucial phylogenetic marker criteria since: (i) it rarely changes relative to speciation events (SCOP domains and FSFs are discovered at average rates of once every ~0.1 and ~5 million years, respectively [27]), (ii) is minimally affected by HGT [37,38], and (iii) is not under active natural selection (structural designs spread at rates of gene duplication and are vastly unaffected by change at sequence level [33]). We caution however that secondary adaptations in organism lifestyles such as parasitism could significantly affect FSF abundances in lineages undergoing reductive evolution. Given all these features, FSFs are well suited for inferring the make up of the urancestor.

Current top-down phylogenetic strategies used to build a tree of life generate unrooted trees and use deep taxa as outgroups *a posteriori *to root phylogenies. One major technical limitation of determining if given genes have an origin in the urancestor using this approach is the need of a universal tree that is accurately rooted. In some traditional studies that were based on sequence conservation, gene genealogies were largely dependent on the position of the root in trees that are used as reference (guide trees)[6,8,9,15]. However, guide trees built from rRNAs or some ancient proteins, such as elongation factors and aminoacyl-tRNA synthases (aRSs), produced rooting scenarios that were not congruent. Inconsistencies of this kind and the possibility of unrecognized paralogy leading to incorrect gene trees have made urancestral gene assignments unreliable [15]. Although a recent technical advance makes it possible to infer ancestral states of a given gene content using the parsimony method (GeneTRACE [49]), it still requires organismal or genomic trees as guides and does not explore the effects of gene abundance. Due to these technical limitations, the first use of FSFs to make inferences of the urancestor was solely based on the distributions of FSFs in genomes (analogous to the *f *index we use here) without any phylogenetic consideration [11].

In contrast, our bottom-up phylogenetic strategy uses the Lundberg method [50] to generate rooted phylogenomic trees without the need of outgroups (Figure 1 and Additional file 1, Figures S1 and S2). Evolution's arrow is established directly by the evolutionary model, the rationale and assumptions of which have been recently reviewed [51]. Operationally, the tree reconstruction algorithm finds the shortest unrooted tree(s) without specifying character polarity and then roots the tree(s) by invoking a hypothetical ancestor defined by ancestral character states and selecting the rooted topology that minimizes overall tree length (see Methods). In this study, a phylogenomic tree that describes the evolution of 420 FL proteomes revealed the three superkingdoms as distinct groups and placed Archaea at the root, with a rooting that was internal (paraphyletic) to the superkingdom (Figure 1A). A tree of life describing the evolution of a balanced set of proteomes corresponding to the three superkingsdoms revealed the same diversification patterns (Additional file 1, Figure S2), suggesting that biases in taxon sampling do not affect the rooting of trees. The archaeal rooting of the tree of life has been reliably obtained in numerous studies with different proteomic sets [27,35,42] and is congruent with phylogenetic analysis of the structure of tRNA [22,52], 5S rRNA [53] and RNase P [54], and of tRNA paralogs [55-58]. While its significance is not the focus and will not be discussed in this paper, a rooting in Archaea (see discussion in [42]) departs significantly from the 'canonical' bacterial rooting of the tree of life, which is traditionally derived from analyses of the sequence of ancient gene paralogs (e.g., ATPases, aaRSs, elongation factors). It thus questions the bacterial-like origin of cellular life inferred from sequence comparisons. In turn, the phylogenomic tree that describes the evolution of 1,420 FSF domain structures showed that the most ancient FSFs at the base of the tree (the *basal_set*) were shared by the three superkingdoms and were mostly universal (Figure 1B). Remarkably, the first loss of ancient FSFs occurred exclusively in archaeal lineages, an observation that also supports the ancestrality of Archaea. Again, patterns of distribution of FSFs in the trees were obtained congruently with numerous proteomic sets and releases of SCOP as genomic sequences and structures were acquired with time [27,35,42,47,59,60].

Trees of proteomes and trees of FSFs are generated from the same genomic structural census but represent two sides of the same story (Figure 1). They describe the evolution of proteomes or the evolution of the FSF structures that make up the potein complement, respectively. Proteomes at the root of the tree of life are populated by FSFs that are shared by all three superkingdoms and proteomes at its crown are enriched in 'signature' FSFs that are unique to individual lineages. Relatively few signature FSFs exist that are specific to superkingdoms. Remarkably, proteome comparisons reveal these signatures are very unequally divided among superkingdoms [61] and already suggest (following parsimony thinking) that bacterial and archaeal lineages evolved from a primordial eukaryotic-like lineage by reductive loss [62]. Phylogenomic analysis confirms this reductive evolutionary tendency, showing that the first diversified lineage to emerge by loss of FSFs gives rise to Archaea, which in turn has the least number of signature FSFs and expresses the lowest levels of diversity and reuse of FSFs in nature [42]. The results we here report confirm once again these patterns (Figure 1B), indicating that the urancestral proteome is populated by ancestral sets of FSFs at the base of the tree of life that appeared in the tree of FSFs before the reductive evolutionary tendency in Archaea was evident [42].

In order to define the proteomic make up of the urancestor, we first identified a set of 352 primitive (plesiomorphic) FSFs (the *352_set*) at the root branch of the tree of FL proteomes. All FSFs of the *352_set *exhibited only gains in genomic abundance, most (314 FSFs) were common to the three superkingdoms, and interestingly, all were present in Archaea (see the four cells that are occupied in the Venn diagram; Figure 2A). However, tracing the *352_set *FSFs in the tree of domain structures revealed the set was not conservative enough to define the urancestor. A timeline describing the age of each FSF unfolded directly from the tree shows that the FSFs that are not universally shared by superkingdoms appeared for the first time in evolution in the order: BE (*nd *= 0.210), AB FSFs (*nd *= 0.415), B (*nd *= 0.433), E (*nd *= 0.538), A (*nd *= 0.538) and AE (*nd *= 0.589) FSF groups (Figure 1B). Remarkably, no FSFs of the ancient BE-specific group were present in the *352_set*, which contains besides the universal *basal_set *the more derived AB, AE and A groups. Similarly, the single A-specific FSF in the set (related to transcriptional regulation; d.236.1) cannot be part of the urancestor (Figure 2A). The FSF is not only absent in Bacteria and Eukarya but is also absent in nearly 50% of archaeal lineages examined (Additional file 1, Table S1). These results suggest many non-universal FSFs in the *352_set *should not be considered urancestral and were the result of the 'modern effect'.

In order to decrease the number of false negatives, FSFs in the *352_set *were assigned as initial characters in a parallel and iterative exercise of tree building and plesiomorphic character selection, with the goal of selecting for the most parsimonious tree of proteomes and the minimum number of FSFs. In each of 30 chains, 50 cycles of iteration dramatically reduced the space of urancestral FSFs (Figure 2A). The iterative procedure resulted in a more realistic *max_set *of 152 FSFs, which: (i) were common to the three superkingdoms (Figure 2A), (ii) excluded FSFs with decreased organismal distribution and genomic abundance levels (i.e with smaller values of *f *and mean of *G*; Figure 2C), and (iii) excluded FSFs that had ambiguous character-state changes in the root branch of the tree of proteomes (Additional file 1, Figure S3). Consequently, the iteration strategy works well to selectively filter false positives assigned to the root branch by the modern effect, decreases biases introduced by the archaeal rooting, and mitigates uncertainties in character-state reconstructions.

We note that real urancestral FSFs that have evolved with intensive losses in numerous proteomic lineages are still possible and their origins will be seen as more derived under the parsimony criterion. These false negatives in the urancestral set cannot be dissected from FSFs that diverged more recently. Since the initial *352_set *includes a significant number of of FSFs in the proteomes examined (~25%), this initial large coverage shields against exclusion of unknown false negatives and makes the *max_set *a maximum bound for the urancestral proteome. On the other hand, false positives resulting from ancient HGT events [6,40] can still occur. For example, FSFs that appeared soon after organismal diversification but transferred extensively to different lineages may be regarded as urancestral. The gap that exists between the discovery of urancestral FSFs and FSFs that emerged at the start of organismal diversification can be identified in the tree of protein domains, since this tree unfolds the evolutionary order of appearances of each of the 1,420 FSFs that are present in the modern proteomes we sampled (Figure 1B). Tracing the *max_set *FSFs in the tree of domain structures revealed that the set was not conservative enough to accurately define the urancestor and that many FSFs had low proteomic distribution (*f) *and abundance (mean of *G*) levels. We therefore defined a more conservative *min_set *by intersecting the *max_set *derived from phylogenetic iteration and the *basal_set *derived from the tree of domain structure (Figure 1B). The *min_set *excluded 82 FSF with relatively smaller *f *and mean of *G *values. This set can be considered a lower bound for the urancestral proteome.

### The proteomic and functional complexity of the urancestor

We here define the urancestor as an entity that accumulated genetic information in a period that spans the emergence of life and the emergence of diversified cellular life. We also consider the urancestor as a primordial isoform of the modern ribonucleoprotein world, regardless of it being a single organism or a communal population [6], especially because it contains fully functional ribosomes (see below). We therefore compare the proteomic and functional sets of the two worlds, the ancient world of the urancestor and the modern world of extant organisms, and make inferences about biological complexity using information in molecules that are modern.

We find that the upper bound urancestral FSF *max_set *contains almost all essential biological processes, including crucial metabolism and transport activities linked to amino acids, nucleotides, carbohydrates, polysaccharides, and coenzymes, and functions associated with the *Information *(*translation*, *DNA replication/repair, transcription, RNA processing*), *Intra-cellular processes *(*transport*, *protein modification*, *proteases*), *Regulation *(e.g. *kinases/phosphatases*, *DNA binding*, *RNA binding*), and *General *(*small molecule binding*, *protein interaction*) categories (Figure 3, Additional file 1, Table S2). As expected, the set lacks the *Extra-cellular processes *category, which includes molecular functions linked to definition of self and inter-cellular interactions (*toxins*, *cell adhesion*, *immunity*, etc). Although some of the sub-categories (i.e. *transcription*, *RNA processing*, and *RNA binding*) were not present in the urancestral *min_set*, the functions of the two urancestral sets are similar and suggest a functional complex entity [8,10]. However, the numbers of urancestral FSFs participating in individual subcategories were always smaller than those of FSFs in modern proteomes (Figure 4, Additional file 1, Table S2). Consequently, the functional repertoire of the urancestor while exhibiting almost all essential functions should be regarded as being simpler than the repertoire of modern proteomes. We suggest FSFs of this limited repertoire acted as melting pot for new molecular functions when organismal lineages emerged, with founder biological activities being primitive and relatively non-specific [19]. The development of the ribosome illustrates such an origin [18].

The numbers of the FSFs in major categories of the *min_set*, especially in *Information*, were smaller than those of the *max_set *(Figure 4). In general, informational genes tend to form multi-component complexes stabilized by protein-protein interactions. For this reason, it has been thought that these genes are refractory to HGTs [63]. The robustness of informational genes against transfer was previously contrasted with the rampant transfer among lineages of ancient metabolic (operational) genes [6,8]. However, a recent study reveals HGT does not exhibit functional preferences and occurs randomly [64]. In turn, analysis of HGT in trees that describe the evolution of function directly from ontological data are congruent with our analysis and suggests a preferential role of HGT in shaping information-related functions [45]. Similarly, a recent comparative statistical analysis of homoplasy levels in trees of proteomes reveals information-related domains at FF level suffered limited but comparatively significant levels of lateral exchange [19]. These FFs were discovered quite early in protein evolution before and after the start of the diversified world. Thus, phylogenomic analysis of HGT of both biological functions and FSF structures can be used to explain why the *min_set *excludes preferentially these ancient HGT-susceptible FSFs. To further examine the role of ancient HGT processes on urancestral proteomic make up, we tested which of 49 functional sub-categories were preferentially enriched in the *max_set *and *min_set *relative to extant FL proteomes. Remarkably, only two functional categories, *translation *(*Information*) and *nucleotide m/tr *(*Metabolism*) were enriched in the *max_set *while metabolism-related *transferases *(*Metabolism*), *small molecule binding *(*General*), and *nucleotide m/tr *(*Metabolism*) functions were enriched in the *min_set *(Table 1). We note that the three sub-categories enriched in the *min_set *include FSF belonging to primordial metabolic folds (see below). While we do not know how many real urancestral FSFs that evolved without major HGT effects were excluded in the *max_set *to *min_set *transition, it is apparent that the proportion of horizontally transferred FSFs in the *min_set *is smaller than that in the *max_set*. Consequently, the absence of *translation *and presence of nucleotide-related metabolic activities in the enriched functions of the *min_set *provides statistical support to operational genes being more robust against ancient HGTs than translation-related informational genes, a result that is in contrast with previous proposals [6,8]. We hypothetize that translation was necessarily simple and flexible during its early metabolic urancestral inception [19]. Fewer molecular interactions between components in a simpler translation system left HGT unchecked and free to shape the spread of FSFs that were recruited for the new translation functions. The numbers of *translation *FSFs are 6 and 34 in the *min_set *and *max_set*, respectively, which represent 6.7% and 38.2% of modern translational FSFs. While ancient HGT processes appear to have shaped the evolution of ancient translation-related genes during urancestral history, HGT may have not affected metabolism to such levels, especially because metabolism was already quite developed when translation materialized in evolution [19]. A detailed phylogenomic analysis of protein domain structure in metabolic networks reveals that the nine most ancient folds were responsible for the explosive appearance of most modern enzymatic functions [36]. A succession of recruitment gateways, each mediated by the discovery of a new fold showed metabolism originated in enzymes of nucleotide metabolism harboring the P-loop-containing NTP hydrolase fold (c.37), probably in pathways linked to the purine metabolic subnetwork [36]. Crucial FSFs of these primordial metabolic folds are part of the urancestral *min_set *and many are part of nucleotide metabolism subnetworks [e.g., P-loop-containing NTP hydrolases (c.37.1); ribulose-phosphate binding barrel (c.1.2); NAD(P)-binding Rossmann-fold domain (c.2.1); ribonuclease H-like (c.55.3)]. The congruent enrichment of *nucleotide m/tr *in both urancestral sets suggests operational genes encoding nucleotide metabolism were built in the urancestor and diverged vertically in primary lineages of the superkingdoms. Interestingly, highly conserved protein-encoding sequences related to nucleotide biosynthetic pathways, including putative phosphoribosyl pyrophosphate synthase and thioredoxin enzymes, were previously identified as being important part of the urancestral set [46]. This is also consistent with a study of physical clustering of genes in bacterial genomes, which also reveals the most ancient group of genes is related to metabolism [65]. Due to statistical limitations of the hypergeometric distribution significant enrichments could not be resolved for the remaining 5 informational and 14 operational categories. A more comprehensive study will be needed to evaluate the extent of HGT in whole sets of ancient operational and informational genes.

Finally, a comparison of structural and functional components of the urancestor and FL proteomes revealed the complexity of the make up of the urancestor relative to extant organisms. In terms of FSF repertoires, the numbers of distinct FSFs (diversity) of the urancestral sets were significantly smaller than those of each and every one of the FL organisms we analyzed (Figure 5A), even if FSF reuse was considered (Figure 5B). Our estimates therefore indicate that the FSF repertoire of the urancestor (70-152 FSFs) and its reuse in domains (303-507 domains) was at least 5 and 3 times smaller than that of extant FL organisms, respectively. Furthermore, the inclusion of artificial urancestral proteomes resurrected *in silico *in tree reconstructions generated trees of proteomes that always placed the urancestor at their base (Figure 6). These results support the distant relationship that exists between the urancestor with the simplest of extant proteomes, confirming the relative simplicity of the reconstructed ancestral entity. Similarly, phylogenetic reconstructions derived from functional data confirm urancestral functions were quantitatively and phylogenetically simpler than functions in any extant FL proteome (Figure 7). Consequently, the actual repertoire of the urancestor inferred from FSFs in modern proteomes, while relatively complex in the number of molecular functions it embodies, is closer to the simple progenote model and distant from the complex cenancestor model. We note however that proteins in the relatively simple FSF repertoire of the urancestor could have been non-specific, harboring a multiplicity of functions. These would have increased the effective complexity of this primordial organism. Furthermore, the complex functional repertoire we reveal suggests the urancestor was a quite advanced version of the progenote, with a multiplicity of metabolic and biosynthetic functions.

### Emergence of translation and ribosomal machinery in the urancestor during the Late Archean

In a previous study, we used phylogenies of FSF and FF to study the emergence of the translation apparatus [19]. Dissection of first appearance of fundamental innovations in molecular machinery (evolutionary landmarks) associated with metabolism and translation revealed translation had metabolic origins. It appeared after the discovery of a large number of metabolic functions but before enzymes necessary for the synthesis of DNA. A clear timeline of molecular diversification was apparent, with domains associated with aminoacylation appearing first, immediately followed by molecular switches and regulatory factors important for tRNA shepherding and RNA transport. Additional file 1, Table S1 shows landmark domains that interact with RNA (some of which have metabolic roles) were present in the *min_set*, including class I (c.26.1, *nd*_FSF _= 0.064) and II catalytic (d.104.1, *nd*_FSF _= 0.128) and anticodon-binding (a.27.1; *nd*_FSF _= 0.141) domains of aRSs, GTP-binding (c.37.1, *nd*_FSF _= 0) and elongation factor (b.43.3, *nd*_FSF _= 0.128) domains of translation factors, and even ribonuclease P and PH domains (d.14.1, *nd*_FSF _= 0.059) crucial for endo- and exoribonucleolytic cleavage of RNA and nucleotydiltransferase activities necessary for damage repair. In contrast, none of domains present in ribonucleotide reductase enzymes responsible for producing the deoxyribonucleotide components necessary for DNA-linked functions, the ferritin-like domain (a.25.1, *nd*_FSF _= 0.242), N-terminal domain of *cbl *(a.48.1, *nd*_FSF _= 0.685), and the the PFL-like glycyl radical enzyme domain (c.7.1, *nd*_FSF _= 0.279), were present in the *min_set*. Only one of these domains, c.7.1, was present in the *max_set*. We note that the reduction of ribonucleotides to deoxyribonucleotides involves the production of an active site thiyl radical that requires contacts with cysteins in all protein domains of the catalytic subunit of the oligomeric enzymatic complex [66], suggesting modern ribonucleotide reductase functions is indeed derived. We also note that the active site domains of class III ribonucleotide reductases share the c.7.1 domain and the associated radical-based chemistry with pyruvate formate-lyase enzymes, a link proposed to have mediated the RNA-to-DNA biological transition [67]. However, phylogenomic analysis at FF level [19] suggests the pyruvate formate-lyase domain (c.7.1.1; *nd*_FF _= 0.518) emerged later than its ribonucleotide reductase counterpart (c.7.1.2; *nd*_FF _= 0.235). It is therefore likely that the urancestor stored genetic information as RNA and not DNA.

The set of FSFs of ribosomal proteins that are universal establish crucial contacts with substructures of the rRNA subunits in the ribosome and appear much later than aRSs and regulatory factors [19]. A careful phylogenetic analysis of ribosomal history directly from protein and RNA structure established the relative time of appearance of ribosomal proteins and rRNA substructures in the ribosome [18]. The study reveals that proteins and RNA co-evolved form the start and structures supporting protein synthesis appeared in a fundamental major transition once processivity functions involving interactions with transfer and templating RNA were already functional. A set of four FSFs were recruited into ribosomal function during this initial period, including ancient ribosomal proteins with OB-fold and related SH3-like small β-barrel folds. Remarkably, all of these FSFs belong exclusively to the urancestral *min_set *(Table 2). A set of 6 additional FSFs that associated with the ribosome immediately after the major transition but before the appearance of the L7/L12 protein complex [18], are all exclusively included in the urancestral *max_set *(Table 2). The L7/L12 complex crucially stimulates the GTPase activity of elongation factor G, a ribosomal factor that catalyzes elongation and enhances ribosomal processivity [68,69]. Primordial protein synthesis was therefore active in the urancestor and the processivity and efficiency of the ribosome was actively improved during urancestral evolution.

In order to place the history of the urancestor and of the ribosome in a timeline, we used a molecular clock of protein domain structure to define evolutionary timescales [20]. Using a clock derived from the tree of FSFs of Figure 1B but using the calibration points of Wang et al. [20], the appearance of the youngest FSFs in the urancestral *min_set *and *max_set *suggests organismal diversification was established sometime between 2.9 and 2 Ga ago (Figure 8). Remarkably, the earliest date coincides with the discovery of arobic metabolism and the start of planet exigenation [20] that lead to the Great Oxidation Event [70], a geological time where oxygen reached 1% of present atmospheric levels. We note that integration of molecular, physiological, paleontological, and geochemical data suggests that a diversified clade of cyanobacteria with marked heterocyst and cell differentiation appeared no later than 2.1 Ga ago [71] and a number of FSFs linked to events of organismal diversification and the fossil record [20] (Figure 8) are only compatible with the existence of an urancestor before that time. This indicates the urancestral *max_set *is not conservative enough to accurately define the urancestor and that the *min_set *is clearly more appropriate. Remarkably, the first and second transitions in ribosomal evolution occurred before the youngest age of *min_set *FSFs, 3.04 and 2.41 Ga ago, respectively, suggesting efficient ribosomal protein synthesis was pre-requisite for organismal diversification and emerged prior to the aerobic metabolism and the start of planet oxygenation (Figure 8).

### The diversification of cellular membranes marks the end of the urancestor

The chirality and chemistry of glycerol membrane lipids is different in Archaea (*sn2,3 *isoprenoid ether lipids) than in Bacteria and Eukarya (*sn1,2 *fatty acid ester lipids), a feature that is claimed to be important for the rise of a diversified organismal world [72-74]. In fact, a widely popular model for organismal diversification is the existence of heterochiral glycerolipids in the primordial membranes of the urancestor, which were synthetized as racemates but then segregated in *sn1,2 *and *sn2,3 *lineages during organismal diversification [75]. These different chiral forms are synthetized in two different metabolic pathways that start with the reduction of a keto group from dihydroxyacetone phosphate (DHAP or glycerone phosphate) and use two different stereochemistry-specific glycerol phosphate backbones. In Bacteria and Eukarya, the synthesis of *sn1,2 *fatty acid ester lipids starts with the convertion of DHAP to sn-glycerol 3-phosphate (G3P) by the activity of glycerol-3-phosphate dehydrogenase (G3PDH)(EC 1.1.1.94). This enzymatic reaction is the first step of pathways needed to produce the ester-fatty acid double layer typical of eukaryotes and mesophylic and psychrophilic bacteria.

In contrast, the first step in the synthesis of *sn2,3 *isoprenoid ether lipids in Archaea starts by the reduction of DHAP to sn-glycerol 1-phosphate (G1P), the enantiomer of G3P, by the Zn^2+^-dependent glycerol-1-phosphate dehydrogenase (G1PDH) metalloenzyme (EC 1.1.1.261). The biosynthesis of polar lipids in Archaea requires the activity of two additional enzymes, the (S)-3-O-geranylgeranylglyceryl phosphate synthase (GGGPS)(EC 2.5.1.41) and the (S)-2,3-di-O-geranylgeranylglyceryl phosphate synthase (DGGGPS)(EC 2.5.1.42) that together alkylate the hydroxy groups of G1P to give *sn-3*-*O*-(geranylgeranyl)glycerol 1-phosphate (GGGP) and 2,3-bis-*O*-(geranylgeranyl)glycerol (DGGGP) and later produce unsaturated archaetidic acid with geranylgeranyl chains and CDP-unsaturated archaeol through downstream activity of the CDP-archaeol synthase (EC 2.7.7.67) enzyme.

To date there are no known exceptions to a G1P backbone chemistry in Archaea and a G3P backbone chemistry in the other two superkingdoms. However, some extremophilic bacteria also contain membrane ether lipids (the typical archaeal trait), including di-glycerol ether lipids, tetraether non-isoprenoid lipids, and mixed ester-ether lipids, but they are all of the *sn1,2 *kind (reviewed in [12]).

Since DGGGPS is not stereospecific, the chirality of the isoprenoid ether lipids in Archaea appears entirely determined by GGGPS [76]. Remarkably, the 6-phosphogluconate dehydrogenase C-terminal domain-like (a.100.1; *nd*_FSF _= 0.110) FSF of the bacterial and eukaryal G3PDH (a.100.1.6; *nd*_FF _= 0.233) and the FMN-linked oxydoreductase domain (c.1.4; *nd*_FSF _= 0.114) FSF of the GGGPS (c.1.4.1; *nd*_FF _= 0.041) necessary for downstream membrane lipid biosynthesis in Archaea were present in the urancestral *min_set *and appeared in the timeline of domain architectures quite early in evolution. A *min_set *G3DPH suggests primordial *sn1,2 *fatty acid ester lipids of some kind were already present in the urancestor. In turn, a *min_set *GGGPS enzyme harboring the only TIM β/α-barrel fold-containing enzyme with a prenyltransferase function [76] suggests chirality-enabling enzymatic activities downstream of G1DPH were already present in the urancestor.

We note that CDP-archaeol synthase activity, which is downstream of G1PDG, does not require specificity for ester or ether bonds nor of glycerol phosphate enantiomers [77] and that full conversion of bacterial-like lipids to archaeal-like lipids requires the crucial discovery or recruitment of G1PDH metalloenzyme activities necessary for the production fo the G1P backbone [76]. The urancestral GGGPS catalyses the first CDP-archaeol biosynthesis pathway-specific step of isoprenoid ether lipids in Archaea but displays a strong preference for the G1P substrate [78]. G1PDH does not yet have a crystallographic structural entry. However, molecular modeling suggests the enzyme was derived from glycerol dehydrogenase (EC 1.1.1.6; e.22.1.2; *nd*_FSF _= 0.288; *nd*_FF _= 0.191) [79], which is present in the three superkingdoms and has the dehydroquinate synthase-like domain (e.22.1) FSF. A total of 110 UniProt entries with EC 1.1.1.261 functions were analyzed with HMMs of structural recognition. All sequence entries were indeed assigned the e.22.1 FSF. Two entries had also the HAD-like domain (c.108.1). While this analysis confirms the original structural assingment [79], we find the G1PDH FSF was not part of any of the urancestral FSF sets. This strongly suggests isoprenoid ether lipids derived from G1P were not present in the urancestor. Instead, and as suggested by Glansdorff et al. [12], *sn1,2 *isoprenoid ether lipids were probably synthetized by the activity of the urancestral GGGPS, which at that time could have exhibited a preference for G3P. Recruitment of a G1PDH in emerging archaeal lineages displaced the possible use of G3P by GGGPS and enabled the synthesis of *sn2,3 *isoprenoid ether lipids using the G1P backbone. It is significant that the molecular clock of FSFs established that the GGGPS c.1.4 FSF appeared 3.27 Ga ago and that the G1PDH e.22.1 FSF appeared 2.45 Ga ago at a time that coincides with the Great Oxidation Event (arrows, Figure 8). In this regard, cyclic and acyclic phytanes and biphytanes, which are present in sediments and petroleum in 2.7-Ga-old metasedimentary rocks in several parts of the world [80,81] and are biomarkers of methanotrophic pelagic microbes, are thought to derive from archaeol and caldarchaeol molecules of Archaea [80]. The existense of GGGPS prior to this date supports the existence of a primordial CDP-archaeol biosynthetic pathway and of ether and ester membrane lipids in the urancestor, prior to the loss of the first FSF in a superkingdom (Archaea) that marks the start of the tripartite world and the earliest date of organismal diversification (~2.9 Ga ago; Figure 8). The synthesis of an enantiomeric alternative of G3P in primordial archaeal lineages, 800 million years later and during late planet oxygenation, provided the proper molecular chirality and backbone necessary for cell membrane diversification. Our findings are important and support the proposal that the urancestor had *sn1,2 *ester and ether fatty acid lipids and that discovery and recruitment of new enzymatic activities resulted in the synthesis of *sn3,4 *isoprenoid ether lipids and the emergence of thermophilic archaeal lineages [12]. Under this scenario, the ester and ether lipids in the urancestor provided already adaptations to adverse conditions (high temperature, pressure, etc), a trend that was later exploited by the emerging Archaeal lineages in a primordial quest towards extremophily.

## Conclusions

The history of proteomes and the protein world was reconstructed from a census of protein domain structure at FSF level of structural complexity in hundreds of FL proteomes. Applying an iterative approach of character state reconstruction, we identified the most parsimonious repertoire of domain structures that was present in the urancestor. Upper and lower bounds of the repertoire defined conservative limits for the diversity of domains and associated functions and showed the uracestor had a functionally complex but relatively simple repertoire, with numbers of FSFs probably orders of magnitude below that of extant organisms. Since the lower bound is more compatible with a molecular clock of FSFs and the fossil record, we can make reasonable inferences about the molecular make up of this primordial organism. The urancestor had an advanced metabolic network, especially rich in nucleotide metabolism enzymes, had primordial pathways for the biosynthesis of membrane glycerol ether and ester lipids, crucial elements of translation, including aRSs, regulatory factors, and a primordial ribosome with protein synthesis capabilities. It lacked however transcription and in advanced evolutionary stages stored genetic information in RNA (not DNA) molecules. As this ancient organism expanded its protein biosynthetic functions ~3 Ga ago, it added crucial ribosomal proteins that enhanced the reliability and processivity of the ribosome and crucial enzymes that diversified its membrane lipid make up. We here propose the enhancement of the primordial ribosomal machinery and the make up of the cellular containment, which coincided with the rise of planetary oxygen, enabled the rise of lineages and a truly diversified world of organisms.

## Methods

### Genomic and proteomic data

We downloaded SUPERFAMILY ver. 1.73 [82] MYSQL database that assigns protein architectures to sequences in the genomes of over 1,000 organisms, 645 of which have been completely sequenced. Manual inspection of organismal lifestyles showed there were 48 archaeal, 239 bacterial, and 133 eukaryotic FL species (420 in total), 71 bacterial and 22 eukaryotic species that were parasitic (93 in total), and 1 archaeal, 111 bacterial, and 20 eukaryotic species that were obligate parasitic (132 in total) in the 645 set. According to our definitions, only one parasitic/obligate parasitic organism was present in Archaea, *Nanoarchaeum equitans*. Given this information, we excluded genomes from all but the 420 FL organisms. SUPERFAMILY has built HMM libraries for SCOP-defined FSFs. Proteomes deposited in the database were searched against the HMM libraries using the iterative Sequence Alignment and Modeling System (SAM) method [83], which has generated FSF assignments covering on average ~ 60% of amino acid residues of individual proteomes [82]. From the local MYSQL, we obtained all FSFs assignments for each of the 645 proteomes. The *E*-value of 10^-4 ^has been known as an ideal cutoff to minimize rate of false positives in the HMM searches [84]. FSF assignments that fulfill the *E*-value cutoff were extracted from the individual proteomes. Domain structures were identified with SCOP *concise classification strings *(*ccs*) (e.g., c.37.1.12, where c represents the protein class, 37 the F, 1 the FSF, and 12 the FF).

### Phylogenomic analysis

In this study, phylogenomic trees describing the evolution of proteomes are reconstructed using a census of the abundance of FSF domains in proteomes [47] instead of simply their occurrence [34]. We have shown that a comparison of the two methods produces phyletic patterns that are congruent [35,42]. Since there has been a hypothetical agreement that genetic components of the urancestor were redundant and versatile [6,85], we chose to build trees of proteomes derived from FSF abundance to identify both the FSF set and the levels of reuse of these FSF domains. This is not possible with an FSF occurrence-based approach.

In SUPERFAMILY, remote homology of protein sequences is determined by more than 30% of sequence identity and by sharing a common ancestor in terms of structure and function [32]. Consequently, a certain FSF can be present in multiple protein sequences. We counted how many times individual FSFs are assigned to each of the proteomes that were sampled. We defined the number of multiple occurrences of a FSF per proteome as a genomic abundance value (*g*) and calculated *g *values for all pair-wise combinations between given proteomes and FSFs, building a two-dimensional data matrix. Empirically, *g *values ranged from 0 to hundreds, resembling morphometic data with a large variance [35,42]. Because existing phylogenetic programs can generally process < 30 phylogenetic character states (depending on user's CPU performance), the space of *g *values in the matrix was reduced to a limited number of character states using gap recoding [86] and the following formula [35].

In this equation, *g*_*ab *_describes the *g *value of FSF *a *in proteome *b *and *g*_max _denotes the maximum *g *value in the matrix. The round function normalizes *g *for particular FSF in a proteome relative to its maximum value, and standardizes values to a 0-23 scale. The 24 transformed values represent character states and were linearly ordered and encoded using an alphanumeric format of numbers 0-9 and letters A-N that are compatible with PAUP* ver. 4.0b10 [44]. The matrix was used to build trees of proteomes by defining taxa and characters as proteomes and FSFs, repectively. The matrix was also transposed to build trees of FSF architectures, with taxa and characters representing FSFs and proteomes, repectively. Trees of proteomes were built by polarizing character states from '0' to 'N' using the ANCSTATES command of the PAUP*, with '0' being primordial. Trees of FSF architectures were built by polarizing states from 'N' to '0', with 'N' being the most ancient. These trees were rooted without invoking outgroup taxa using the Lundberg method [50]; the most ancient proteome or architecture was positioned at the base of their corresponding trees. Our evolutionary model considers that the abundance and diversity of individual FSF architectures increases progressively in nature, even when expanding FSFs suffer loss in individual lineages, and even when FSFs are selectively or differentially constrained during evolution. Consequently, we consider ancient architectures are more abundant and widely present in the protein world than younger ones, supporting the polarization from 'N' to '0'. We also assume that proteomes have built their FSF repertoires progressively in evolution, increasing both the diversity and abundance of the FSF make up. Genomes that are ancient developed their repertoires earlier from a pool of FSF architectures that was comparatively simpler. Consequently, their repertoires are today simpler than those that developed their repertoires more recently from a more complex and diverse pool. This supports the '0' to 'N' polarization. We note that convergent evolution (e.g. due to HGT) and rampant expansion of genes by gene or genome duplications in specific lineages can bias architectural complexity, especially in ancient organisms. While the extent of these effects remains controversial [6,8], convergent evolution of domains appears to be rare [37,38] and the effect of HGT to be limited at this high levels of hierarchical complexity [39,45,64]. Moreoever, trees of proteomes match for the most part established organismal classification (e.g., [35,42]). We therefore postulate that these evolutionary mechanisms do not overwhelm the vertical inheritance of protein architectures along individual organismal lineages. Details on character argumentation and absence of circularity in assumptions were comprehensively discussed in previous publications and are not developed further here [35,36,42,47].

Phylogenomic trees were reconstructed from the transformed and polarized matrices using the maximum parsimony method in PAUP* with 1,000 replicates of random taxon addition, tree bisection reconnection (TBR) branch swapping, and maxtrees unrestricted. The trees generated were rooted by the Lundberg method, which does not require the need of an outgroup taxon. Phylogenetic confidence was evaluated by the nonparametric bootstrap method with 1,000 replicates (resampling size matches the number of the genomes sampled; TBR; maxtrees, unrestricted). The consensus of most parsimonious proteome trees was obtained using the Python library SumTrees with the option of 50% majority-rule [87]. The degree of phylogenetic signal for taxa was measured using the skewness (*g*_*1*_) test with a tree length distribution obtained from 1,000 random trees [43].

Since trees are rooted and are highly unbalanced, we unfolded the relative age of protein domains directly for each phylogeny as a distance in nodes (node distance, *nd*) from the hypothetical ancestral architecture at the base of the trees in a relative 0-1 scale. Given a rooted tree, we calculated *nd *by counting the number of internal nodes along a lineage from the root to a terminal node (a leaf) of the tree on a relative 0-1 scale with the following equation: *nd*_*a *_= (# of internal nodes between nodes *r *and *a*)/(# of internal nodes between nodes *r *and *m*), where *a *means a target leaf node, *r *is a hypothetical root node, and *m *is a leaf node that has the largest possible number of internal nodes from the node *r*. Consequently, the *nd *value of the most ancestral taxon is 0 while that of the most recent one is 1. *nd *can be a good measure of age given a rooted tree since the emergence of protein domains (i.e. taxa) is displayed by their ability to diverge (cladogenesis or molecular speciation) rather than by the amount of character state change that exists in branches of the tree (branch lengths).

### Iteration analysis

Phylogenetic characters (FSFs) defining the root of the tree of proteomes can be used to identify the urancestral repertoire of protein domains. However, FSFs appearing after the advent of the diversified world (the 'modern effect') were by definition absent in the urancestor and must be excluded to avoid bias in the identification of urancestral FSFs. We developed an iteration analysis to refine the urancestral set of FSFs. First, we extracted FSFs positioned at the root branch of the tree of proteomes, which was reconstructed prior to each step of iteration. As an initial character set, the FSFs were imported into each of 30 chains, each of which proceeded with the following steps: (i) reconstruct a proteome tree; (ii) extract FSFs that are in the root branch; (iii) update the character set with the FSFs; (iv) iterate steps 1 to 3 a total of 50 times.

We then gathered all of the most parsimonious trees that were generated from the 1,500 iterations, and selected one tree with minimal tree length and the smallest set of FSF domains. Prior to iteration analysis, simulation with hundreds of iterations per chain showed that decreases of tree length and the number of the plesiomorphic FSFs become stationary after ~40 iterations. FSF sets in the root branches were progressively stabilized as iterations progressed in individual chains and ambiguous plesiomorphic FSF assignments were reduced. In addition, multiple chains made it possible to explore as many parsimony islands that were possible to avoid the risk of the search of optimal trees being trapped by sub-optimal portions of a given tree space [88].

### Functional annotations of FSF domain architectures

Nearly 80% of FSFs defined in SCOP have more than one family [89]. Because a single family can be regarded as a functionally orthologous unit [30], FSFs with more than one family can be considered multi-functional. For example, the P-loop-containing NTP hydrolase (c.37.1) is the most abundant FSF in nature and has more than 20 distinct families, most of which are involved in tens of metabolic pathways [36]. In spite of the difficulty of assigning functions to FSFs, Vogel and Chothia [41] suggested a coarse-grained functional classification for known FSFs based on information in various resources, including COG and GO databases, and literature surveys. When an FSF had multiple families, the most dominant function was assigned to the FSF. While this coarse-grained classification cannot provide a complete functional profile for individual FSFs, the classification is highly suitable to infer the originating functions of FSFs because the most ancient function of a FSF tends to be the most abundant and widely present in contemporary proteomes. For urancestral FSFs uncovered by the iteration analysis, we annotated their function using the coarse-grained functional classification. Although it is likely that functional recruitments or take-overs had arisen during evolution of the urancestral lineage, we do not consider the role of these processes. Their dissection is per se a difficult problem.

### Enrichment of functional categories

In the coarse-grained classification of functions we described above, FSFs are assigned to 50 sub-categories that are subsequently grouped into seven major categories. The sub-category named "not annotated" was not considered in this study. We evaluated which functional categories were enriched by the set (sample) of urancestral FSFs in comparison to the set of all FSFs (background) of the sampled proteomes. For each of the two sets, we counted the number of FSFs that were annotated to individual sub-categories, and then calculated the probability of enrichment of a particular sub-category present in urancestral FSFs using the hypergeometric distribution and the equation:

Observed values *M *and *k *indicate the number of FSFs that are assigned to a sub-category in the background and sample, respectively. The values *N *and *n *are the total number of FSFs of the two sets regardless of the functional classification. The probability *P(X = k) *implies the chance that a random variable *X *has *k *FSFs for a given functional category. Referring to the equation and previous literature [38], we calculated *P *values for the individual sub-categories that have *k/n *larger than *M/N*, and evaluated statistical significance with 95% confidence level (*P *< 0.05).

### Assigining domain structures to KEGG enzymes

Enzymes that are related to metabolic pathways of glycerol cell membrane lipids were identified using the Kyoto Encyclopedia of Genes and Genomes (KEGG; http://www.genome.jp/kegg/). We retrieved UniProt Knowledgebase Identifiers (UniProtKB IDs) from KEGG, the Enzyme Commission (EC) numbers of the enzymes. Protein sequences that correspond to the UniProt IDs were obtained from the UniProt (http://www.uniprot.org/). To assign SCOP protein domains at the FSF level of classification, we searched the protein sequences of the individual enzymes against HMMs of total FSFs that are provided by the locally downloaded SUPERFAMILY database. The FSFs were assigned to given protein sequences using the SAM method with an *E*-value cutoff of 10^-4^.

## Abbreviations

FSF: fold superfamily; FF: fold family; FL: free living; Ga: giga-annum; HGT: horizontal gene transfer; LUCA: last universal common ancestor; SCOP: Structural Classification of Proteins.

## Competing interests

The authors declare that they have no competing interests.

## Authors' contributions

Both authors design experiments, analyzed data, and wrote the paper. Both authors read and approved the final manuscript.

## Supplementary Material

Additional file 1 **Figure S1. Inclusion of non-FL proteomes in phylogenomic analyses makes relationships of lineages incorrect**. Cladogram of a most parsimonious rooted tree obtained from 1,446 FSFs and 645 proteomes (1,414 parsimoniously informative sites; 177,229 steps; CI = 0.057; RI = 0.780; *g*_1 _= -0.065). Terminal leaves of Archaea (A), Bacteria (B), and Eukarya (E) were labeled in red, blue, and cyan, respectively. The dotted lines explicitly display the borders between two superkingdoms. The life-styles of proteomes were displayed using a vertical bar beside their terminal leaves. Free-living (FL), parasitic (O), and obligate parasitic (OP) proteomes were labeled in blue, gray, and red, respectively. The 645 proteomes consist of 420 FL (48 A, 239 B, 133 E), 93 parasitic (0 A, 71 B, 22 E), and 132 obligate parasitic (1 A, 111 B, 20 E) organisms. OP lineages were present at the base of the three superkingdoms. **Figure S2. Representative phylogenomic tree of proteomes describing the evolution of 102 FL organisms sampled equally across superkingdoms (34 archaeal, bacterial, and eukaryal proteomes, respectively)**. One most parsimonious tree was reconstructed based on genomic abundances of 1,370 FSFs in the proteomes (1,311 parsimoniously informative sites; 50,564 steps; CI = 0.194; RI = 0.724; *g*_1 _= -0.486). Non-parametric bootstrap values that have more than 50% supports were shown above or below branches that cluster the superkingdoms or much higher groups. Terminal leaves of Archaea, Bacteria, and Eukarya were labeled in red, blue, and cyan, respectively. **Figure S3. The iterative analysis increases the reliability of FSFs that are positioned in the root nodes of the proteome trees**. For the chain that produced the minimum number of LUCA FSFs and the smallest tree length, the ambiguity of character-state changes for the FSFs in the root nodes of the proteome trees was examined. In the plot, the x-axis indicates the number of iterations from 1 to 50, with zero representing the initial proteome tree. The y-axis denotes the number of the FSFs that had ambiguous character-state changes in their root branches of the proteome trees. The dramatic decreases of ambiguous character-state changes in root branches were consistently observed in all of the 30 chains that were examined. **Table S1. FSF repertoires in the *352_set *and urancestral *max_set *and *min_set*. Table S2. FSF molecular functions. Table S3. Functional enrichment**. Click here for file

## References

[B1] Woese CR Kandler O Wheelis ML Towards a natural system of organisms: proposal for the domains Archaea, Bacteria, and Eucarya Proc Natl Acad Sci USA 1990 87 4576 4579 10.1073/pnas.87.12.4576 2112744 PMC54159

[B2] Woese CR Fox GE Phylogenetic structure of the prokaryotic domain: the primary kingdoms Proc Natl Acad Sci USA 1977 74 5088 5090 10.1073/pnas.74.11.5088 270744 PMC432104

[B3] May RM How many species are there on earth? Science 1988 241 1441 1449 10.1126/science.241.4872.1441 17790039

[B4] Bull AT Goodfellow M Slater JH Biodiversity as a source of innovation in biotechnology Annu Rev Microbiol 1992 46 219 252 10.1146/annurev.mi.46.100192.001251 1444255

[B5] Whitman WB Coleman DC Wiebe WJ Prokaryotes: the unseen majority Proc Natl Acad Sci USA 1998 95 6578 6583 10.1073/pnas.95.12.6578 9618454 PMC33863

[B6] Woese CR The universal ancestor Proc Natl Acad Sci USA 1998 95 6854 6859 10.1073/pnas.95.12.6854 9618502 PMC22660

[B7] Kyrpides N Overbeek R Ouzounis C Universal protein families and the functional content of the last universal common ancestor J Mol Evol 1999 49 413 423 10.1007/PL00006564 10485999

[B8] Penny D Poole A The nature of the last universal common ancestor Curr Opin Genet Dev 1999 9 672 677 10.1016/S0959-437X(99)00020-9 10607605

[B9] Koonin EV Comparative genomics, minimal gene-sets and the last universal common ancestor Nat Rev Microbiol 2003 1 127 136 10.1038/nrmicro751 15035042

[B10] Ouzounis CA Kunin V Darzentas N Goldovsky L A minimal estimate for the gene content of the last universal common ancestor-exobiology from a terrestrial perspective Res Microbiol 2006 157 57 68 10.1016/j.resmic.2005.06.015 16431085

[B11] Ranea JAG Sillero A Thornton JM Orengo CA Protein superfamily evolution and the last universal common ancestor (LUCA) J Mol Evol 2006 63 513 525 10.1007/s00239-005-0289-7 17021929

[B12] Glansdorff N Xu Y Labedan B The last universal common ancestor: emergence, constitution and genetic legacy of an elusive forerunner Biol Direct 2008 3 29 10.1186/1745-6150-3-29 18613974 PMC2478661

[B13] Bandea C A unifying scenario on the origin and evolution of cellular and viral domains Nature Proceedings 2009 http://hdl.handle.net/10101/npre.2009.3888.1

[B14] Forterre P Giant viruses: Conflicts in revisiting the virus concept Intervirology 2010 53 362 378 10.1159/000312921 20551688

[B15] Gribaldo S Brochier C Phylogeny of prokaryotes: does it exist and why should we care? Res Microbiol 2009 160 513 521 10.1016/j.resmic.2009.07.006 19631737

[B16] Theobald DL A format test of the theory of universal common ancestry Nature 2010 465 219 222 10.1038/nature09014 20463738

[B17] Forterre P Philippe H Where is the root of the universal tree of life? BioEssays 1999 21 871 879 10.1002/(SICI)1521-1878(199910)21:10<871::AID-BIES10>3.0.CO;2-Q 10497338

[B18] Harish A Caetano-Anollés G Ribosomal history reveals origins of modern protein synthesis 2011 in press 10.1371/journal.pone.0032776 PMC3299690 22427882

[B19] Caetano-Anollés D Kim KM Mittenthal JM Caetano-Anollés G Proteome evolution and the metabolic origins of translation and cellular life J Mol Evol 2011 72 14 32 10.1007/s00239-010-9400-9 21082171

[B20] Wang M Jiang Y-Y Kim KM Qu G Ji HF-Mittenthal JE Zhang HY-Caetano-Anollés G A universal molecular clock of folds and its power in tracing the early history of aerobic metabolism and planet oxygenation Mol Biol Evol 2011 28 567 582 10.1093/molbev/msq232 20805191

[B21] Reanney DC On the origin of prokaryotes Theor Biol 1974 48 243 251 10.1016/0022-5193(74)90194-5 4376201

[B22] Sun F-J Caetano-Anollés G Evolutionary patterns in the sequence and structure of transfer RNA: early origins of Archaea and viruses PLoS Comput Biol 2008 4 e1000018 10.1371/journal.pcbi.1000018 18369418 PMC2265525

[B23] Edgell DR Doolittle WF Archaea and the origin(s) of DNA replication proteins Cell 1997 89 995 998 10.1016/S0092-8674(00)80285-8 9215620

[B24] Olsen GJ Woese CR Lessons from an archaeal genome: What are we learning from *Methanococcus jannaschii*? Trends Genet 1996 12 377 379 10.1016/0168-9525(96)30092-9 8909123

[B25] Bult CJ White O Olsen GJ Zhou L Fleischmann RD Sutton GG Blake JA FitzGerald LM Clayton RA Gocayne JD Complete genome sequence of the methanogenic archaeon, *Methanococcus jannaschii* Science 1996 273 1058 1073 10.1126/science.273.5278.1058 8688087

[B26] Fitch WM Upper K The phylogeny of tRNA sequences provides evidence for ambiguity reduction in the origin of the genetic code Cold Spring Harb Symp Quant Biol 1987 52 759 767 3454288 10.1101/sqb.1987.052.01.085

[B27] Caetano-Anollés G Wang M Caetano-Anollés D Mittenthal JE The origin, evolution and structure of the protein world Biochemical J 2009 417 621 637 10.1042/BJ20082063 19133840

[B28] Kim KM Sung S Caetano-Anollés G Han JY Kim H An approach of orthology detection from homologous sequences under minimum evolution Nucleic Acids Res 2008 36 e110 10.1093/nar/gkn485 18676448 PMC2553584

[B29] Morrison DA Why would phylogenetics ignore computerized sequence alignment? Syst Biol 2009 58 150 158 10.1093/sysbio/syp009 20525575

[B30] Murzin A Brenner SE Hubbard T Chothia C SCOP: a structural classification of proteins for the investigation of sequences and structures J Mol Biol 1995 247 536 540 7723011 10.1006/jmbi.1995.0159

[B31] Liolios K Mavrommatis K Tavernarakis N Kyrpides NC The genome on line database (GOLD) in 2007: status of genomic and metagenomic projects and their associated metadata Nucleic Acids Res 2007 36 D475 D479 10.1093/nar/gkm884 17981842 PMC2238992

[B32] Gough J Karplus K Hughey R Chothia C Assignment of homology to genome sequences using a library of hidden Markov models that represent all proteins of known structure J Mol Biol 2001 313 903 919 10.1006/jmbi.2001.5080 11697912

[B33] Illergård K Ardell DH Elofsson A Structure is three to ten times more conserved than sequence - a study of structural response in protein cores Proteins 2009 77 499 508 10.1002/prot.22458 19507241

[B34] Yang S Doolittle RF Bourne PE Phylogeny determined based on protein domain content Proc Natl Acad Sci USA 2005 102 373 378 10.1073/pnas.0408810102 15630082 PMC540256

[B35] Wang M Caetano-Anollés G Global phylogeny determined by the combination of protein domains in proteomes Mol Biol Evol 2006 23 2444 2454 10.1093/molbev/msl117 16971695

[B36] Caetano-Anollés G Kim HS Mittenthal JE The origin of modern metabolic networks inferred from phylogenomic analysis of protein architecture Proc Natl Acad Sci USA 2007 104 9358 9363 10.1073/pnas.0701214104 17517598 PMC1890499

[B37] Gough J Convergent evolution of domain architectures (is rare) Bioinformatics 2005 21 1464 1471 10.1093/bioinformatics/bti204 15585523

[B38] Forslund K Henricson A Hollich V Sonnhammer ELL Domain tree-based analysis of protein architecture evolution Mol Biol Evol 2007 25 254 264 18025066 10.1093/molbev/msm254

[B39] Yang S Bourne PE The evolutionary history of proteins domains viewed by species phylogeny PLoS One 2009 4 e8378 10.1371/journal.pone.0008378 20041107 PMC2794708

[B40] Brown JR Ancient horizontal gene transfer Nat Rev Genet 2003 4 121 132 12560809 10.1038/nrg1000

[B41] Vogel C Chothia C Protein family expansions and biological complexity PLoS Comput Biol 2006 2 e48 10.1371/journal.pcbi.0020048 16733546 PMC1464810

[B42] Wang M Yafremava LS Caetano-Anollés D Mittenthal JE Caetano-Anollés G Reductive evolution of architectural repertoires in proteomes and the birth of the tripartite world Genome Res 2007 17 1572 1585 10.1101/gr.6454307 17908824 PMC2045140

[B43] Hillis DM Huelsenbeck JP Signal, noise, and reliability in molecular phylogenetic analyses J Hered 1992 83 189 195 1624764 10.1093/oxfordjournals.jhered.a111190

[B44] Swofford DL Phylogenetic analysis using parsimony and other program (PAUP*), ver. 4.0b10 2002 Sinauer, Sunderland, MA

[B45] Kim KM Caetano-Anollés G Emergence and evolution of modern molecular functions inferred from phylogenomic analysis of ontological data Mol Biol Evol 2010 27 1710 1733 10.1093/molbev/msq106 20418223

[B46] Delaye L Becerra A Lazcano A The last common ancestor: What's in a name? Orig Life Evol Biosph 2005 35 537 554 10.1007/s11084-005-5760-3 16254691

[B47] Caetano-Anollés G Caetano-Anollés D An evolutionarily structured universe of protein architecture Genome Res 2003 13 1563 1571 10.1101/gr.1161903 12840035 PMC403752

[B48] Caetano-Anollés G Caetano-Anollés D Universal sharing patterns in proteomes and evolution of protein fold architecture and life J Mol Evol 2005 60 484 498 10.1007/s00239-004-0221-6 15883883

[B49] Kunin V Ouzounis CA GeneTRACE - reconstruction of gene content of ancestral species Bioinformatics 2003 19 1412 1416 10.1093/bioinformatics/btg174 12874054

[B50] Lundberg JG Wagner networks and ancestors Syst Zool 1972 18 1 32

[B51] Caetano-Anollés G Sun FJ Wang M Yafremava LS Harish A Kim HS Knudsen V Caetano-Anollés D Mittenthal JE Origins and evolution of modern biochemistry: insights from genomes and molecular structure Front Biosci 2008 13 5212 5240 18508583 10.2741/3077

[B52] Sun F-J Caetano-Anollés G The origin and evolution of tRNA infrerred from phylogenetic analysis of structure J Mol Evol 2008 66 21 35 10.1007/s00239-007-9050-8 18058157

[B53] Sun F-J Caetano-Anollés G The evolutionary history of the structure of 5S ribosomal RNA J Mol Evol 2009 69 430 443 10.1007/s00239-009-9264-z 19639237

[B54] Sun F-J Caetano-Anollés G The ancient history of the structure of ribonuclease P and the early origins of Archaea BMC Bioinformatics 2010 11 153 10.1186/1471-2105-11-153 20334683 PMC2858038

[B55] Xue H Tong K-L Marck C Grosjean H Wong JT-F Transfer RNA paralogs: evidence for genetic code-amino acid biosynthesis coevolution and an archaeal root of life Gene 2003 310 59 66 12801633 10.1016/s0378-1119(03)00552-3

[B56] Xue H Ng S-K Tong K-L Wong JT-F Congruence of evidence for a Methanopyrus-proximal root of life based on transfer RNA and aminoacyl-tRNA synthetase genes Gene 2005 360 120 130 10.1016/j.gene.2005.06.027 16153784

[B57] Wong JT-F Chen J Mat W-K Ng S-K Xue H Polyphasic evidence delineating the root of life and roots of biological domains Gene 2007 403 39 52 10.1016/j.gene.2007.07.032 17884304

[B58] Di Giulio M The tree of life might be rooted in the branch leading to Nanoarchaeota Gene 2007 401 108 113 10.1016/j.gene.2007.07.004 17689206

[B59] Wang M Boca SM Kalelkar R Mittenthal JE Caetano-Anollés G A phylogenomic reconstruction of the protein world based on a genomic census of protein fold architecture Complexity 2006 12 27 40 10.1002/cplx.20141

[B60] Wang M Caetano-Anollés G The evolutionary mechanics of domain organization in proteomes and the rise ofmodularity in the protein world Structure 2009 17 66 78 10.1016/j.str.2008.11.008 19141283

[B61] Kurland CG Canbäck B Berg OG The origins of modern proteomes Biochimie 2007 89 1454 1463 10.1016/j.biochi.2007.09.004 17949885

[B62] Kurland CG Collins LJ Penny D Genomics and the irreducible nature of eukaryotic cells Science 2006 312 1011 1016 10.1126/science.1121674 16709776

[B63] Jain R Rivera MC Lake JA Horizontal gene transfer among genomes: The complexity hypothesis Proc Natl Acad Sci USA 1999 96 3801 3806 10.1073/pnas.96.7.3801 10097118 PMC22375

[B64] Choi I-G Kim SH Global extent of horizontal gene transfer Proc Natl Acad Sci USA 2007 104 4489 4494 10.1073/pnas.0611557104 17360551 PMC1815472

[B65] Danchin A Fang G Noria S The extant core bacterial proteome is an archive of the origin of life Proteomics 2007 7 875 889 10.1002/pmic.200600442 17370266

[B66] Uhlin U Eklund H Structure of ribonucleotide reductase protein R1 Nature 1994 370 533 539 10.1038/370533a0 8052308

[B67] Stubbe J Ribonucleotide reductases: the link between an RNA and a DNA world? Curr Op Struct Biol 2000 10 731 736 10.1016/S0959-440X(00)00153-6 11114511

[B68] Rodnina MV Savelsbergh A Katunin VI Wintermeyer W Hydrolysis of GTP by elongation factor G drives tRNA movement on the ribosome Nature 1997 385 37 41 10.1038/385037a0 8985244

[B69] Savelsbergh A Mohr D Wilden B Wintermeyer W Rodnina MV Stimulation of the GTPase activity of translation elongation factor G by ribosomal protein L7/12 J Biol Chem 2000 275 890 894 10.1074/jbc.275.2.890 10625623

[B70] Sessions AL Doughty DM Welander PV Summons RE Newman DK The continuing puzzle of the great oxidation event Curr Biol 2009 19 R567 R574 10.1016/j.cub.2009.05.054 19640495

[B71] Tomitani A Knoll AH Cavanaugh CM Ohno T The evolutionary diversification of cyanobacteria: molecular-phylogenetic and paleontological perspectives Proc Natl Acad Sci USA 2006 103 5442 5447 10.1073/pnas.0600999103 16569695 PMC1459374

[B72] Wächtershäuser G Groundworks for an evolutionary biochemistry: the iron-sulphur world Prog Biophys Mol Biol 1992 58 85 201 10.1016/0079-6107(92)90022-X 1509092

[B73] Koga Y Kyuragi T Nishihara M Sone N Did archaeal and bacterial cells arise independently from noncelullar precursors? A hypothesis stating that the advent of membrane phospholipids with enantiomeric glycerophosphate backbones caused the separation of the two lines of descent J Mol Evol 1998 46 54 63 10.1007/PL00006283 9419225

[B74] Peretó J López-Garcia P Moreira D Ancestral lipid biosynthesis and early membrane evolution Trends Biochem Sci 2004 29 469 477 10.1016/j.tibs.2004.07.002 15337120

[B75] Wächtershäuser G From pre-cells to eukarya: a tale of two lipids Mol Microbiol 2003 47 13 22 12492850 10.1046/j.1365-2958.2003.03267.x

[B76] Payandeh J Pai EF Enzyme-driven speciation: crystallizing Archaea via lipid capture J Mol Evol 2007 64 364 374 10.1007/s00239-006-0141-8 17253090

[B77] Morii H Nishimara M Koga Y CTP: 2,3-di-O-geranylgeranyl-sn-glycero-1-phosphate cytidyltransferase in the methanogenic archaeon *Methanothermobacter thermoautotrophicus* J Biol Chem 2000 275 36568 36574 10.1074/jbc.M005925200 10960477

[B78] Chen A Zhang D Poulter CD (S)-geranylgeranylglyceryl phosphate synthase. Purification and characterization of the first pathway-specific enzyme in archaebacterial membrane lipid biosynthesis J Biol Chem 1993 268 21701 217055 8408023

[B79] Daiyasu H Hiroike T Koga Y Toh H Analysis of membrane stereochemistry with homology modeling of sn-glycerol-1-phosphate dehydrogenase Prot Eng 2002 15 987 995 10.1093/protein/15.12.987 12601138

[B80] Ventura GT Kenig F Reddy CM Schieber J Frysinger GS Nelson RK Dinel E Gaines RB Schaeffer P Molecular evidence of Late Archean archaea and the presence of a subsurface hydrothermal biosphere Proc Natl Acad Sci USA 2007 104 14260 14265 10.1073/pnas.0610903104 17726114 PMC1964827

[B81] Fischer WW Life before the rise of oxygen Nature 2008 455 1051 1052 10.1038/4551051a 18948942

[B82] Wilson D Pethica R Zhou Y Talbot C Vogel C Madera M Chothia C Gough J SUPERFAMILY-sophisticated comparative genomics, data mining, visualization and phylogeny Nucleic Acids Res 2009 37 D380 D386 10.1093/nar/gkn762 19036790 PMC2686452

[B83] Gough J Chothia C SUPERFAMILY: HMMs representing all proteins of known structure. SCOP sequence searches, alignments, and genome assignments Nucleic Acids Res 2002 30 268 272 10.1093/nar/30.1.268 11752312 PMC99153

[B84] Wilson D Madera M Vogel C Chothia C Gough J The SUPERFAMILY database in 2007: families and functions Nucleic Acids Res 2007 35 D308 D313 10.1093/nar/gkl910 17098927 PMC1669749

[B85] Labedan B Boyen A Baetens M Charlier D Chen P Cunin R Durbeco V Glansdorff N Herve G Legrain C The evolutionary history of carbamoyltransferases: a complex set of paralogous genes was already present in the last universal common ancestor J Mol Evol 1999 49 461 473 10.1007/PL00006569 10486004

[B86] Thiele K The holy grail of the perfect character: The cladistic treatment of morphometric data Cladistics 1993 9 275 304 10.1111/j.1096-0031.1993.tb00226.x 34929957

[B87] Sukumaran J Holder MT SumTrees: summarization of split support on phylogenetic trees. version 10.0.2 2008 (part of the DendroPy phylogenetic computation library version 2.1.3);

[B88] Nixon KC The parsimony ratchet, a new method for rapid parsimony analysis Cladistics 1999 15 407 414 10.1111/j.1096-0031.1999.tb00277.x 34902938

[B89] Andreeva A Howorth D Chandonia J-M Brenner SE Hubbard TJP Chothia C Murzin AG Data growth and its impact on the SCOP database: new developments Nucleic Acids Res 2008 36 D419 D425 18000004 10.1093/nar/gkm993 PMC2238974

