# Supplementary material for: The proteomic complexity and rise of the primordial ancestor of diversified life
Source: BMC Evol Biol. 2011 May 25;11:140. doi: 10.1186/1471-2148-11-140 (PMC3123224; doi:10.1186/1471-2148-11-140)
Supplement: Additional file 1 — Figure S1. Inclusion of non-FL proteomes in phylogenomic analyses makes relationships of lineages incorrect. Cladogram of a most parsimonious rooted tree obtained from 1,446 FSFs and 645 proteomes (1,414 parsimoniously informative sites; 177,229 steps; CI = 0.057; RI = 0.780; g1 = -0.065). Terminal leaves of Archaea (A), Bacteria (B), and Eukarya (E) were labeled in red, blue, and cyan, respectively. The dotted lines explicitly display the borders between two superkingdoms. The life-styles of proteomes were displayed using a vertical bar beside their terminal leaves. Free-living (FL), parasitic (O), and obligate parasitic (OP) proteomes were labeled in blue, gray, and red, respectively. The 645 proteomes consist of 420 FL (48 A, 239 B, 133 E), 93 parasitic (0 A, 71 B, 22 E), and 132 obligate parasitic (1 A, 111 B, 20 E) organisms. OP lineages were present at the base of the three superkingdoms. Figure S2. Representative phylogenomic tree of proteomes describing the evolution of 102 FL organisms sampled equally across superkingdoms (34 archaeal, bacterial, and eukaryal proteomes, respectively). One most parsimonious tree was reconstructed based on genomic abundances of 1,370 FSFs in the proteomes (1,311 parsimoniously informative sites; 50,564 steps; CI = 0.194; RI = 0.724; g1 = -0.486). Non-parametric bootstrap values that have more than 50% supports were shown above or below branches that cluster the superkingdoms or much higher groups. Terminal leaves of Archaea, Bacteria, and Eukarya were labeled in red, blue, and cyan, respectively. Figure S3. The iterative analysis increases the reliability of FSFs that are positioned in the root nodes of the proteome trees. For the chain that produced the minimum number of LUCA FSFs and the smallest tree length, the ambiguity of character-state changes for the FSFs in the root nodes of the proteome trees was examined. In the plot, the x-axis indicates the number of iterations from 1 to 50, with zero representing the initial pr [file 1471-2148-11-140-S1.PDF]

Additional data file

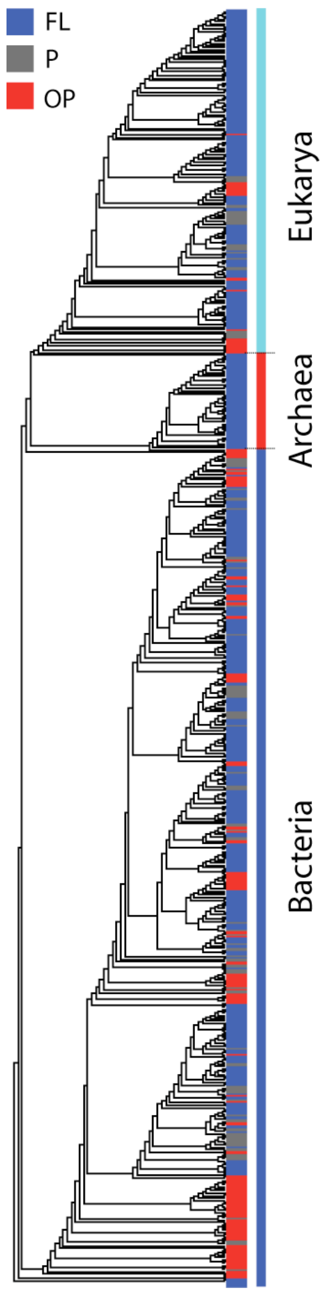

Figure S1. Inclusion of non-FL proteomes in phylogenomic analyses makes relationships of lineages incorrect. Cladogram of a most parsimonious rooted tree obtained from 1,446 FSFs and 645 proteomes (1,414 parsimoniously informative sites; 177,229 steps; CI = 0.057; RI = 0.780; g_1 = -0.065). Terminal leaves of Archaea (A), Bacteria (B), and Eukarya (E) were labeled in red, blue, and cyan, respectively. The dotted lines explicitly display the borders between two superkingdoms. The life-styles of proteomes were displayed using a vertical bar beside their terminal leaves. Free-living (FL), parasitic (O), and obligate parasitic (OP) proteomes were labeled in blue, gray, and red, respectively. The 645 proteomes consist of 420 FL (48 A, 239 B, 133 E), 93 parasitic (0 A, 71 B, 22 E), and 132 obligate parasitic (1 A, 111 B, 20 E) organisms. OP lineages were present at the base of the three superkingdoms.

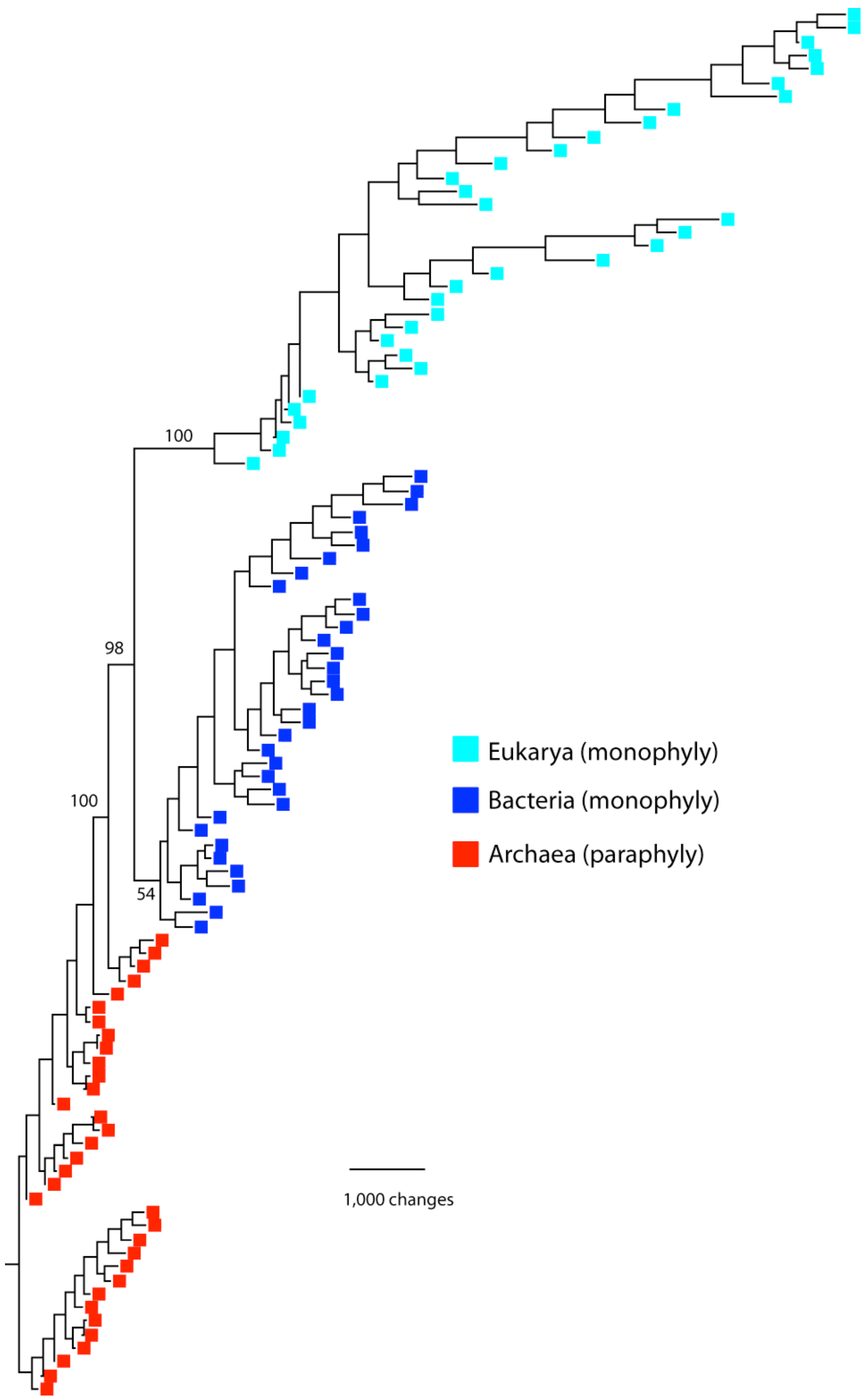

Figure S2. Representative phylogenomic tree of proteomes describing the evolution of 102 FL organisms sampled equally across superkingdoms (34 archaeal, bacterial, and eukaryal proteomes, respectively). One most parsimonious tree was reconstructed based on genomic abundances of 1,370 FSFs in the proteomes (1,311 parsimoniously informative sites; 50,564 steps; CI = 0.194; RI = 0.724; g_1 = -0.486). Non-parametric bootstrap values that have more than 50% supports were shown above or below branches that cluster the superkingdoms or much higher groups. Terminal leaves of Archaea, Bacteria, and Eukarya were labeled in red, blue, and cyan, respectively.

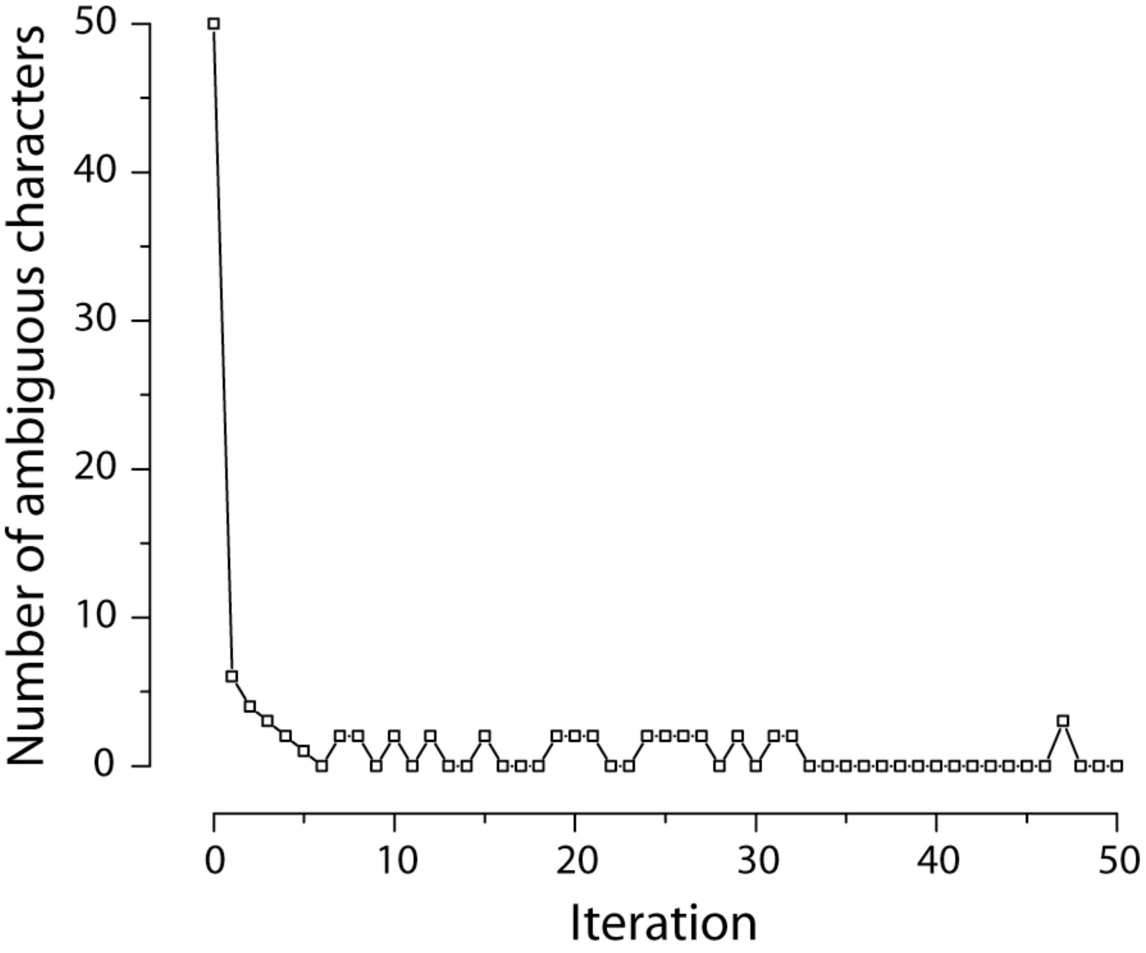

Figure S3. The iterative analysis increases the reliability of FSFs that are positioned in the root nodes of the proteome trees. For the chain that produced the minimum number of LUCA FSFs and the smallest tree length, the ambiguity of character-state changes for the FSFs in the root nodes of the proteome trees was examined. In the plot, the x-axis indicates the number of iterations from 1 to 50, with zero representing the initial proteome tree. The y-axis denotes the number of the FSFs that had ambiguous character-state changes in their root branches of the proteome trees. The dramatic decreases of ambiguous character-state changes in root branches were consistently observed in all of the 30 chains that were examined.

Table S1. FSF repertoires in the *352_set* and urancestral *max_set* and *min_set*. The table provides SCOP functional assignments of FSFs, mean of *G* and *f* values, and taxonomic distribution in superkingdoms. FSFs of the *352_set* were grouped into sets of 70 (*min_set*), 82 (unique to *max_set*), and 200 FSFs (not present in urancestral sets). f_T, *f* value for the three superkingdoms; f_A, *f* value for Archaea; f_B, *f* value for Bacteria; f_E, *f* value for Eukarya; DS, FSF distribution in superkingdoms (see text).

Only in *352_set* (200 FSFs)

FSF	SCOP functional description	Functional subcategories	Functional categories	Mean of G	f_T	f_A	f_B	f_E	DS
d.162.1	LDH C-terminal domain-like	Redox	Metabolism	2.95	91.9	79.2	90.4	100	ABE
c.91.1	PEP carboxykinase-like	Energy	Metabolism	4.28	97.6	85.4	100	98.5	ABE
d.78.1	RPB5-like RNA polymerase subunit	Transcription	Information	0.88	39.9	97.9	0	91	AE
d.284.1	PurS-like	Nucleotide m/tr	Metabolism	1.8	87.6	77.1	90.8	86.5	ABE
a.137.1	Ribosomal protein L39e	Translation	Information	0.77	34.4	91.7	0	75.9	ABE
a.76.1	Iron-dependent repressor protein, dimerization domain	DNA-binding	Regulation	0.82	33.7	85.4	42.3	0	AB
d.58.48	MTH1187/YkoF-like	Unknown function	Other	0.83	39	62.5	42.7	24.1	ABE
d.58.43	Mechanosensitive channel protein MscS (YggB), C-terminal domain	Transport	Processes_IC	1.9	62.5	70.8	92.5	6	ABE
d.58.40	D-ribose-5-phosphate isomerase (RpiA), lid domain	Carbohydrate m/tr	Metabolism	1.59	74.6	100	59.8	92.5	ABE
a.151.1	Glutamyl tRNA-reductase dimerization domain	Translation	Information	1.11	53.2	75	72.4	11.3	ABE
b.43.4	Riboflavin synthase domain-like	Coenzyme m/tr	Metabolism	5.09	96.7	77.1	99.2	100	ABE
d.264.1	Prim-pol domain	Unknown function	Other	1.08	48.5	100	13	94	ABE
a.143.1	RPB6/omega subunit-like	DNA-binding	Regulation	1.86	91	97.9	90	91	ABE
c.57.1	Molybdenum cofactor biosynthesis proteins	Coenzyme m/tr	Metabolism	3.34	94.3	95.8	94.1	94.7	ABE
b.1.13	Superoxide reductase-like	Redox	Metabolism	0.32	14.5	50	14.2	2.3	ABE
d.235.1	FYSH domain	Unknown function	Other	0.97	41.8	100	0	96.2	AE
c.23.6	Cobalamin (vitamin B12)-binding domain	Coenzyme m/tr	Metabolism	2.28	73.6	75	90	44.4	ABE
c.23.4	Succinyl-CoA synthetase domains	Coenzyme m/tr	Metabolism	3.64	91.2	97.9	85.4	100	ABE
c.23.8	N5-CAIR mutase (phosphoribosylaminoimidazole carboxylase, PurE)	Nucleotide m/tr	Metabolism	2.23	96.2	91.7	100	91.7	ABE
d.201.1	SRP19	Transport	Processes_IC	0.87	41.1	97.9	0	94.7	AE
d.58.20	NAD-binding domain of HMG-CoA reductase	Coenzyme m/tr	Metabolism	1.2	52.7	100	21.3	92.5	ABE
a.110.1	Aldehyde ferredoxin oxidoreductase, C-terminal domains	Redox	Metabolism	0.54	18.5	68.8	18.8	0	AB
c.92.2	Helical backbone metal receptor	General	General	3.02	72.2	100	98.3	15.8	ABE
c.92.1	Chelatase	Other enzymes	Metabolism	2.39	91.4	75	91.6	97.7	ABE
d.58.39	Glutamyl tRNA-reductase catalytic, N-terminal domain	Other enzymes	Metabolism	1.23	59.1	85.4	78.2	15.8	ABE
a.24.13	Domain of the SRP/SRP receptor G-proteins	Signal transduction	Regulation	2.95	98.6	100	98.7	98.5	ABE
a.24.16	Nucleotidyltransferase substrate binding subunit/domain	Nucleotide m/tr	Metabolism	1.73	56.1	54.2	74.1	24.8	ABE
d.143.1	SAICAR synthase-like	Nucleotide m/tr	Metabolism	3.2	98.6	89.6	100	100	ABE
d.15.10	TGS-like	Ligand binding	General	3.3	98.1	85.4	100	100	ABE
d.168.1	Succinate dehydrogenase/fumarate reductase flavoprotein, catalytic domain	E- transfer	Metabolism	2.96	92.9	83.3	93.3	96.2	ABE
a.60.9	lambda integrase-like, N-terminal domain	DNA-binding	Regulation	2.53	70.3	75	97.9	19.5	ABE
a.2.5	Prefoldin	Protein modification	Processes_IC	2.03	44.4	100	2.9	99.2	ABE
a.2.2	Ribosomal protein L29 (L29p)	Translation	Information	2.05	95.5	97.9	98.3	90.2	ABE
d.13.1	HIT-like	Carbohydrate m/tr	Metabolism	3.86	99	93.8	100	100	ABE

a.4.1	Homeodomain-like	DNA-binding	Regulation	8.87	96.7	77.1	99.2	100	ABE
a.4.2	Methylated DNA-protein cysteine methyltransferase, C-terminal domain	Transferases	Metabolism	2.23	88.8	97.9	96.7	72.2	ABE
d.15.4	2Fe-2S ferredoxin-like	Redox	Metabolism	4.73	96.4	91.7	95.8	100	ABE
c.132.1	Bacterial fluorinating enzyme, N-terminal domain	Unknown function	Other	0.44	21.6	68.8	24.3	0	AB
d.91.1	N-terminal domain of eukaryotic peptide chain release factor subunit 1, ERF1	Translation	Information	0.94	42.3	100	0	97.7	AE
f.35.1	Multidrug efflux transporter AcrB transmembrane domain	Transport	Processes_IC	5.54	96.4	89.6	97.9	97	ABE
d.87.1	FAD/NAD-linked reductases, dimerisation (C-terminal) domain	Redox	Metabolism	4.02	98.6	93.8	99.2	100	ABE
d.58.21	Molybdenum cofactor biosynthesis protein C, MoaC	Coenzyme m/tr	Metabolism	1.75	83.6	95.8	86.6	74.4	ABE
g.41.9	RNA polymerase subunits	DNA replication/repair	Information	1.14	41.3	100	0	94.7	AE
a.36.1	Signal peptide-binding domain	Signal transduction	Regulation	2.09	98.1	100	97.9	98.5	ABE
c.62.1	vWA-like	Cell adhesion	Processes_EC	4.9	91.9	85.4	89.1	100	ABE
c.64.1	Pyruvate-ferredoxin oxidoreductase, PFOR, domain III	Redox	Metabolism	1.79	60.3	100	66.5	35.3	ABE
c.117.1	Amidase signature (AS) enzymes	Other enzymes	Metabolism	3.42	93.8	75	94.6	100	ABE
c.113.1	HemD-like	Coenzyme m/tr	Metabolism	2.03	89.3	85.4	90.4	89.5	ABE
d.58.1	4Fe-4S ferredoxins	Redox	Metabolism	5.08	97.9	100	97.1	99.2	ABE
d.58.2	Aspartate carbamoyltransferase, Regulatory-chain, N-terminal domain	Nucleotide m/tr	Metabolism	0.34	16.6	95.8	9.6	0.8	ABE
d.58.5	GlnB-like	Signal transduction	Regulation	3.1	96.9	95.8	99.6	93.2	ABE
d.58.4	Dimeric alpha+beta barrel	Secondary metabolism	Metabolism	4.14	88.1	97.9	93.7	75.2	ABE
d.58.6	Nucleoside diphosphate kinase, NDK	Nucleotide m/tr	Metabolism	2.64	94.5	97.9	92.9	97	ABE
b.153.1	PheT/TilS domain	Unknown function	Other	1.78	82.2	56.3	98.7	62.4	ABE
d.29.1	Ribosomal protein L31e	Translation	Information	0.95	40.6	100	0	92.5	AE
f.23.28	Preprotein translocase SecE subunit	Transport	Processes_IC	0.83	37.1	83.3	0	87.2	AE
d.58.16	PAP/Archaeal CCA-adding enzyme, C-terminal domain	Transcription	Information	1.05	42.3	100	0	97.7	AE
d.58.10	Acylphosphatase/BLUF domain-like	Kinases/phosphatases	Regulation	1.91	73.6	93.8	74.5	65.4	ABE
d.58.12	eEF-1beta-like	Translation	Information	1.03	39	100	0	87.2	AE
c.47.1	Thioredoxin-like	Redox	Metabolism	8.84	99.8	97.9	100	100	ABE
c.47.2	RNA 3'-terminal phosphate cyclase, RPTC, insert domain	Other enzymes	Metabolism	0.58	27.6	68.8	9.6	45.1	ABE
d.129.1	TATA-box binding protein-like	Transcription	Information	2.2	65.8	100	40.2	100	ABE
c.55.5	Nitrogenase accessory factor-like	Unknown function	Other	0.88	29	79.2	35.1	0	AB
c.55.2	Creatinase/prolidase N-terminal domain	Lipid m/tr	Metabolism	2.93	97.6	95.8	97.1	100	ABE
d.280.1	Sulfolobus fructose-1,6-bisphosphatase-like	Other enzymes	Metabolism	0.25	12.6	77.1	6.7	0	AB
d.282.1	SSo0622-like	NONA	NONA	0.59	27.1	54.2	0	66.2	AE
a.1.2	alpha-helical ferredoxin	Redox	Metabolism	4.32	97.1	100	97.1	97	ABE
b.53.1	Ribosomal protein L25-like	Translation	Information	2.56	95	100	91.6	100	ABE
b.45.1	FMN-binding split barrel	Coenzyme m/tr	Metabolism	3.85	95.5	95.8	95	97	ABE
b.122.1	PUA domain-like	RNA binding, m/tr	Regulation	3.81	99.3	100	99.2	100	ABE
e.19.1	HydA/Nqo6-like	Other enzymes	Metabolism	2.2	78.4	100	76.2	75.2	ABE
c.71.1	Dihydrofolate reductase-like	Coenzyme m/tr	Metabolism	2.78	94.1	87.5	99.2	88	ABE
d.61.1	LigT-like	Other enzymes	Metabolism	2.16	83.8	100	77.4	90.2	ABE

c.90.1	Tetrapyrrole methylase	Transferases	Metabolism	3.65	98.8	97.9	100	97.7	ABE
d.17.6	Pre-PUA domain	RNA binding, m/tr	Regulation	0.95	40.6	97.9	0	93.2	AE
d.179.1	Substrate-binding domain of HMG-CoA reductase	Coenzyme m/tr	Metabolism	1.15	50.1	100	15.9	94	ABE
b.49.3	Aminopeptidase/glucanase lid domain	Other enzymes	Metabolism	0.74	29.5	79.2	36	0	AB
d.177.1	FAH	Other enzymes	Metabolism	2.95	87.6	85.4	82.4	98.5	ABE
a.94.1	Ribosomal protein L19 (L19e)	Translation	Information	0.92	39.7	100	0	89.5	AE
a.74.1	Cyclin-like	DNA-binding	Regulation	3.3	44.4	100	2.5	100	ABE
b.36.1	PDZ domain-like	Signal transduction	Regulation	5.84	98.8	93.8	100	99.2	ABE
e.50.1	AF1104-like	NONA	NONA	1.15	44.2	68.8	10.5	96.2	ABE
c.56.1	HybD-like	Other enzymes	Metabolism	0.96	34.9	66.7	48.1	0	AB
d.95.2	Homing endonucleases	DNA replication/repair	Information	0.84	30.9	35.4	38.9	15	ABE
d.241.1	Translation initiation factor 2 beta, aIF2beta, N-terminal domain	Translation	Information	1.25	42.8	100	0	99.2	AE
d.208.1	MTH1598-like	Unknown function	Other	0.75	35.4	95.8	12.6	54.9	ABE
d.261.1	Hypothetical protein PH1602	Unknown function	Other	1.18	54.9	100	43.9	58.6	ABE
d.206.1	YggU-like	Unknown function	Other	1.04	48.9	52.1	42.3	60.2	ABE
d.270.1	2-isopropylmalate synthase LeuA, allosteric (dimerisation) domain	Other enzymes	Metabolism	1.89	74.1	77.1	89.5	45.9	ABE
c.34.1	Homo-oligomeric flavin-containing Cys decarboxylases, HFCD	Secondary metabolism	Metabolism	2.61	97.4	95.8	100	94	ABE
d.210.1	Argininosuccinate synthetase, C-terminal domain	Other enzymes	Metabolism	1.97	92.9	87.5	95.8	90.2	ABE
c.26.3	UDP-glucose/GDP-mannose dehydrogenase C-terminal domain	Energy	Metabolism	2.24	84.1	81.3	86.6	81.2	ABE
b.137.1	Rof/RNase P subunit-like	RNA binding, m/tr	Regulation	0.8	38.5	89.6	0	89.5	AE
d.152.1	Aldehyde ferredoxin oxidoreductase, N-terminal domain	Redox	Metabolism	0.55	18.8	68.8	19.2	0	AB
c.111.1	Activating enzymes of the ubiquitin-like proteins	Coenzyme m/tr	Metabolism	3.79	96	97.9	93.7	100	ABE
d.50.2	Porphobilinogen deaminase (hydroxymethylbilane synthase), C-terminal domain	Other enzymes	Metabolism	1.88	89.8	85.4	91.2	89.5	ABE
b.141.1	Bacterial fluorinating enzyme, C-terminal domain	Unknown function	Other	0.44	21.6	68.8	24.3	0	AB
c.129.1	MoCo carrier protein-like	Other enzymes	Metabolism	2.37	81.2	75	99.6	51.1	ABE
d.41.5	Molybdopterin synthase subunit MoaE	Coenzyme m/tr	Metabolism	1.63	77	89.6	75.7	75.2	ABE
c.6.2	Glycoside hydrolase/deacetylase	Other enzymes	Metabolism	3.89	92.9	72.9	95	97	ABE
c.6.3	PHP domain-like	Other enzymes	Metabolism	3.17	95.7	89.6	99.2	92.5	ABE
a.7.3	Succinate dehydrogenase/fumarate reductase flavoprotein C-terminal domain	Redox	Metabolism	2.53	89.5	83.3	88.7	94	ABE
a.5.8	Hypothetical protein AF0491, middle domain	Unknown function	Other	0.86	41.6	100	0	95.5	AE
a.5.6	Double-stranded DNA-binding domain	DNA-binding	Regulation	0.81	39.4	100	0	88.7	AE
c.131.1	Peptidyl-tRNA hydrolase II	Other enzymes	Metabolism	1.08	43.5	100	4.2	94	AE
f.20.1	Clc chloride channel	Ion m/tr	Processes_IC	2.5	77.4	62.5	69.5	97.7	ABE
c.125.1	Creatininase	Other enzymes	Metabolism	0.63	27.3	64.6	33.5	3	ABE
f.22.1	ABC transporter involved in vitamin B12 uptake, BtuC	Transport	Processes_IC	2.46	66.7	95.8	97.1	2.3	ABE
c.8.2	LeuD/IlvD-like	Energy	Metabolism	3.23	96.7	93.8	95.8	100	ABE
c.8.3	Carbamoyl phosphate synthetase, small subunit N-terminal domain	Coenzyme m/tr	Metabolism	2.23	96.7	85.4	98.7	97.7	ABE
c.8.1	Phosphohistidine domain	Kinases/phosphatases	Regulation	2.24	74.6	97.9	97.5	25.6	ABE
c.77.1	Isocitrate/Isopropylmalat	Redox	Metabolism	4.42	99.3	93.8	100.4	100	ABE

	e dehydrogenase-like								
a.111.1	Acid phosphatase/Vanadium-dependent haloperoxidase	Redox	Metabolism	3.65	90.5	66.7	90.4	100	ABE
a.43.1	Ribbon-helix-helix	DNA-binding	Regulation	1.24	46.1	95.8	61.1	1.5	ABE
d.79.3	L30e-like	Translation	Information	4.02	99.3	100	99.2	100	ABE
b.85.4	dUTPase-like	Nucleotide m/tr	Metabolism	2.4	91.7	97.9	89.1	94.7	ABE
b.85.6	MoeA C-terminal domain-like	Coenzyme m/tr	Metabolism	2.12	82.7	89.6	84.9	76.7	ABE
c.104.1	YjeF N-terminal domain-like	Unknown function	Other	2.08	91	100	86.2	97	ABE
d.75.1	tRNA-intron endonuclease N-terminal domain-like	Translation	Information	0.28	11.6	95.8	0	2.3	AE
c.106.1	SurE-like	Unknown function	Other	1.4	62.2	75	71.1	42.1	ABE
d.59.1	Ribosomal protein L30p/L7e	Translation	Information	2.33	93.3	100	88.7	100	ABE
c.88.1	Glutaminase/Asparaginase	Amino acids m/tr	Metabolism	1.73	72.7	100	62.8	81.2	ABE
a.4.11	RNA polymerase subunit RPB10	RNA processing	Information	0.77	37.1	100	0	81.2	AE
a.92.1	Carbamoyl phosphate synthetase, large subunit connection domain	Amino acids m/tr	Metabolism	2.24	96	85.4	98.3	96.2	ABE
d.161.1	ADC synthase	Other enzymes	Metabolism	2.38	80.3	89.6	95.4	50.4	ABE
a.183.1	Nop domain	RNA binding, m/tr	Regulation	1.3	42.8	100	0	99.2	AE
d.101.1	Ribonuclease PH domain 2-like	Nucleotide m/tr	Metabolism	3.06	92.2	72.9	96.2	92.5	ABE
c.82.1	ALDH-like	Redox	Metabolism	5.85	99	91.7	100	100	ABE
c.50.1	Macro domain-like	Proteases	Processes_IC	3.06	92.6	66.7	94.6	99.2	ABE
c.52.2	tRNA-intron endonuclease catalytic domain-like	Translation	Information	1.17	41.3	100	0	94.7	AE
c.52.1	Restriction endonuclease-like	DNA replication/repair	Information	3	94.8	100	91.2	100	ABE
e.12.1	DNA topoisomerase IV, alpha subunit	DNA replication/repair	Information	1.06	47	93.8	11.3	94.7	ABE
e.22.1	Dehydroquinate synthase-like	Redox	Metabolism	3.21	98.1	100	99.2	96.2	ABE
a.80.1	DNA polymerase III clamp loader subunits, C-terminal domain	DNA replication/repair	Information	3.25	97.1	97.9	95.8	100	ABE
a.130.1	Chorismate mutase II	Amino acids m/tr	Metabolism	1.69	71.7	87.5	81.6	48.9	ABE
c.14.1	ClpP/crotonase	Proteases	Processes_IC	6.45	98.6	89.6	100	100	ABE
b.40.1	Staphylococcal nuclease	Nucleotide m/tr	Metabolism	1.72	61	45.8	43.1	99.2	ABE
b.40.5	Inorganic pyrophosphatase	Other enzymes	Metabolism	1.85	74.6	66.7	62.8	99.2	ABE
e.18.1	HydB/Nqo4-like	Other enzymes	Metabolism	2.21	80	100	76.6	79.7	ABE
b.34.1	C-terminal domain of transcriptional repressors	Transcription	Information	1.44	54.4	79.2	79.9	0	AB
a.60.4	Rad51 N-terminal domain-like	DNA replication/repair	Information	1.89	68.2	100	53.1	84.2	ABE
a.60.8	HRDC-like	DNA replication/repair	Information	1.98	80.8	100	69.9	94	ABE
d.269.1	BtrG-like	Unknown function	Other	1.94	64.4	33.3	59	85.7	ABE
d.64.1	eIF1-like	Translation	Information	1.74	59.4	97.9	29.3	100	ABE
d.156.1	S-adenosylmethionine decarboxylase	Coenzyme m/tr	Metabolism	1.44	61.5	64.6	44.8	91	ABE
c.81.1	Formate dehydrogenase/DMSO reductase, domains 1-3	Redox	Metabolism	3.2	88.4	95.8	88.7	85.7	ABE
e.7.1	Carbohydrate phosphatase	Carbohydrate m/tr	Metabolism	3.7	94.5	93.8	92.1	100	ABE
a.127.1	L-aspartase-like	Amino acids m/tr	Metabolism	3.89	99.5	95.8	100.4	100	ABE
c.1.22	UROD/MetE-like	Coenzyme m/tr	Metabolism	2.87	94.5	97.9	92.9	97	ABE
c.1.20	tRNA-guanine transglycosylase	Translation	Information	2.2	90.5	97.9	93.3	83.5	ABE
c.1.21	Dihydropteroate synthetase-like	Coenzyme m/tr	Metabolism	2.85	92.6	97.9	98.3	81.2	ABE
d.258.1	Chorismate synthase, AroC	Other enzymes	Metabolism	1.65	81	91.7	96.2	50.4	ABE
d.96.1	Tetrahydrobiopterin biosynthesis enzymes-like	Nucleotide m/tr	Metabolism	3.57	97.9	93.8	98.7	98.5	ABE
d.273.1	YjbQ-like	Unknown function	Other	1.42	62	83.3	61.9	54.9	ABE

d.90.1	FMN-dependent nitroreductase-like	E- transfer	Metabolism	3.07	88.6	85.4	96.7	75.9	ABE
f.34.1	Mechanosensitive channel protein MscS (YggB), transmembrane region	Transport	Processes_IC	1.97	67.5	83.3	94.6	13.5	ABE
f.44.1	Ammonium transporter	Transport	Processes_IC	2.85	92.2	72.9	92.1	100	ABE
f.14.1	Voltage-gated potassium channels	Ion m/tr	Processes_IC	4.21	86.9	75	82.4	100	ABE
c.27.1	Nucleoside phosphorylase/phosphoribosyltransferase catalytic domain	Nucleotide m/tr	Metabolism	2.3	88.1	95.8	97.9	68.4	ABE
g.59.1	Zinc-binding domain of translation initiation factor 2 beta	Ion binding	General	1.21	42.8	100	0	99.2	AE
c.25.1	Ferredoxin reductase-like, C-terminal NADP-linked domain	Redox	Metabolism	4.64	95	79.2	95.8	100	ABE
a.25.1	Ferritin-like	Ion m/tr	Processes_IC	4.76	99.8	100	100	100	ABE
c.33.1	Isochorismatase-like hydrolases	Other enzymes	Metabolism	2.99	90.3	77.1	89.1	97.7	ABE
a.118.16	Translin	RNA processing	Information	0.96	35.6	66.7	2.5	84.2	ABE
g.41.8	Zn-binding ribosomal proteins	Translation	Information	1.79	42.3	100	0	97.7	AE
g.41.1	Methionyl-tRNA synthetase (MetRS), Zn-domain	Ion binding	General	1.39	66.5	97.9	50.2	85	ABE
g.41.7	Aspartate carbamoyltransferase, Regulatory-chain, C-terminal domain	Other regulatory function	Regulation	0.35	17.1	95.8	10.5	0.8	ABE
c.1.3	Thiamin phosphate synthase	Coenzyme m/tr	Metabolism	1.99	79.8	83.3	95.4	51.1	ABE
d.31.1	Cdc48 domain 2-like	Small molecule binding	General	1.47	45.4	100	4.6	99.2	ABE
c.72.3	CoaB-like	Other enzymes	Metabolism	2.02	97.9	95.8	99.2	97	ABE
c.9.2	Ribosomal protein L32e	Translation	Information	0.93	39.4	100	0	88.7	AE
a.204.1	all-alpha NTP pyrophosphatases	Other enzymes	Metabolism	0.88	38.2	47.9	35.6	39.8	ABE
e.10.1	Prokaryotic type I DNA topoisomerase	DNA replication/repair	Information	2.59	99.5	97.9	100	100	ABE
c.76.1	Alkaline phosphatase-like	Other enzymes	Metabolism	4.99	98.3	100	97.5	100	ABE
c.74.1	AraD-like aldolase/epimerase	Other enzymes	Metabolism	2.58	86.5	85.4	82	95.5	ABE
d.126.1	Pentein	General	General	2.36	80.3	97.9	67.4	97.7	ABE
c.42.1	Arginase/deacetylase	Amino acids m/tr	Metabolism	3.86	92.6	100	87.4	100	ABE
d.283.1	Putative modulator of DNA gyrase, PmbA/TldD	DNA replication/repair	Information	1.56	48.2	89.6	65.3	3	ABE
b.103.1	MoeA N-terminal region -like	Coenzyme m/tr	Metabolism	2.18	84.3	95.8	86.6	76.7	ABE
b.84.2	Rudiment single hybrid motif	Transport	Processes_IC	4.11	99.3	95.8	100	100	ABE
c.48.1	TK C-terminal domain-like	Transferases	Metabolism	4.49	99.5	100	99.6	100	ABE
c.16.1	Lumazine synthase	Coenzyme m/tr	Metabolism	1.67	77.7	87.5	94.6	44.4	ABE
b.52.2	ADC-like	General	General	3.82	94.8	100	91.6	99.2	ABE
a.46.2	Nucleoside phosphorylase/phosphoribosyltransferase N-terminal domain	Nucleotide m/tr	Metabolism	2.1	79.6	95.8	97.5	42.1	ABE
a.160.1	PAP/OAS1 substrate-binding domain	RNA processing	Information	1.85	43.5	100	0.8	100	ABE
b.80.4	Alpha subunit of glutamate synthase, C-terminal domain	Other enzymes	Metabolism	1.86	81	72.9	89.5	69.2	ABE
d.68.4	YhbY-like	Unknown function	Other	0.95	41.1	68.8	52.7	10.5	ABE
d.68.5	C-terminal domain of ProRS	Unknown function	Other	1.11	49.6	97.9	15.9	93.2	ABE
d.68.6	AlbA-like	DNA-binding	Regulation	0.89	30.4	81.3	0	66.9	AE
b.82.5	TRAP-like	Transcription	Information	1.01	41.8	68.8	41.8	32.3	ABE
b.82.1	RmlC-like cupins	Nitrogen m/tr	Metabolism	5.71	99.3	100	99.2	100	ABE
d.58.51	eIF-2-alpha, C-terminal domain	Translation	Information	0.88	41.3	100	0	94.7	AE
d.236.1	DNA-binding protein Tfx	DNA-binding	Regulation	0.11	5.5	47.9	0	0	A
e.26.1	Prismane protein-like	E- transfer	Metabolism	0.93	34.9	47.9	33.1	33.8	ABE

d.230.1	N-terminal, heterodimerisation domain of RBP7 (RpoE)	Protein interaction	General	1.16	42.8	100	0	99.2	AE
f.40.1	V-type ATP synthase subunit C	E- transfer	Metabolism	1.06	45.4	93.8	5.9	99.2	ABE
c.1.17	Nicotinate/Quinolinate PRTase C-terminal domain-like	Nucleotide m/tr	Metabolism	1.71	80.3	79.2	88.7	66.2	ABE
c.1.15	Xylose isomerase-like	Carbohydrate m/tr	Metabolism	3.26	93.1	100	91.2	94.7	ABE
c.83.1	Aconitase iron-sulfur domain	Energy	Metabolism	3.27	96.7	93.8	95.8	100	ABE
d.110.7	Roadblock/LC7 domain	Unknown function	Other	1.04	33	33.3	19.7	57.1	ABE

Only in *max_set* (82 FSFs)

FSF	SCOP functional description	Functional subcategories	Functional categories	Mean of G	f_T	f_A	f_B	f_E	DS
a.97.1	An anticodon-binding domain of class I aminoacyl-tRNA synthetases	Translation	Information	2.03	92.4	72.9	99.6	87.2	ABE
d.58.42	N-utilization substance G protein NusG, N-terminal domain	Other regulatory function	Regulation	1.53	69.4	77.1	100	12	ABE
b.51.1	ValRS/IleRS/LeuRS editing domain	Translation	Information	3.56	99.8	100	100	100	ABE
a.7.12	PhoU-like	Receptor activity	Regulation	2.02	68.2	100	97.9	3.8	ABE
c.120.1	PIN domain-like	Unknown function	Other	4.58	100	100	100	100	ABE
d.141.1	Ribosomal protein L6	Translation	Information	3.29	98.6	100	99.2	97.7	ABE
a.4.7	Ribosomal protein L11, C-terminal domain	Translation	Information	2.4	98.8	100	99.6	97.7	ABE
d.58.26	GHMP Kinase, C-terminal domain	Kinases/phosphatases	Regulation	3.29	99.5	100	99.6	100	ABE
c.12.1	Ribosomal proteins L15p and L18e	Translation	Information	2.66	99.8	100	100	100	ABE
c.107.1	DHH phosphoesterases	Other enzymes	Metabolism	2.84	94.5	100	96.2	90.2	ABE
d.28.1	Ribosomal protein S19	Translation	Information	2.2	98.1	100	99.2	96.2	ABE
c.101.1	Undecaprenyl diphosphate synthase	Secondary metabolism	Metabolism	2.53	99.5	100	100	99.2	ABE
d.58.15	Ribosomal protein S10	Translation	Information	2.4	98.8	100	99.2	98.5	ABE
e.29.1	beta and beta-prime subunits of DNA dependent RNA-polymerase	Transcription	Information	3.84	99.5	100	99.6	100	ABE
d.54.1	Enolase N-terminal domain-like	Other enzymes	Metabolism	3.35	99	97.9	99.6	99.2	ABE
d.56.1	GroEL-intermediate domain like	Protein modification	Processes_IC	3.6	99.8	100	100	100	ABE
d.129.2	Phosphoglucomutase, C-terminal domain	Carbohydrate m/tr	Metabolism	3.31	99.5	100	100	99.2	ABE
c.55.4	Translational machinery components	Translation	Information	3.59	99.5	100	100	99.2	ABE
d.139.1	PurM C-terminal domain-like	Other enzymes	Metabolism	4.08	99.5	100	99.6	100	ABE
d.52.3	Prokaryotic type KH domain (KH-domain type II)	Translation	Information	3.61	99.3	100	100	98.5	ABE
a.129.1	GroEL equatorial domain-like	Protein modification	Processes_IC	3.71	100	100	100	100	ABE
d.131.1	DNA clamp	DNA replication/repair	Information	3.42	99.8	100	100	100	ABE
c.51.1	Class II aaRS ABD-related	Translation	Information	4.12	100	100	100.4	100	ABE
c.51.4	ITPase-like	Unknown function	Other	3.11	99.8	100	100	100	ABE
e.13.1	DNA primase core	DNA replication/repair	Information	1.79	76.5	100	100	26.3	ABE
a.69.1	C-terminal domain of alpha and beta subunits of F1 ATP synthase	Energy	Metabolism	3.28	99.5	100	100	99.2	ABE
b.49.1	N-terminal domain of alpha and beta subunits of F1 ATP synthase	Transport	Processes_IC	3.16	99.3	97.9	100	99.2	ABE
d.77.1	Ribosomal protein L5	Translation	Information	2.21	98.6	100	100	96.2	ABE
a.75.1	Ribosomal protein S7	Translation	Information	2.32	99	100	99.6	98.5	ABE
d.15.9	Glutamine synthetase, N-terminal domain	Coenzyme m/tr	Metabolism	2.71	95.5	100	94.6	96.2	ABE
c.80.1	SIS domain	Energy	Metabolism	4.15	99.8	100	100	100	ABE
b.87.1	LexA/Signal peptidase	Transcription	Information	3.5	98.1	85.4	100	99.2	ABE

d.74.4	GAD domain	Translation	Information	1.67	82.2	83.3	99.2	51.9	ABE
d.74.3	RBP11-like subunits of RNA polymerase	Transcription	Information	2.75	99.8	100	100	100	ABE
b.15.1	HSP20-like chaperones	Protein modification	Processes_IC	4	94.3	100	90.4	100	ABE
e.52.1	NAD kinase	Other enzymes	Metabolism	2.5	98.1	100	98.7	97	ABE
c.56.2	Purine and uridine phosphorylases	Nucleotide m/tr	Metabolism	3.43	99	100	99.2	99.2	ABE
c.20.1	Initiation factor IF2/eIF5b, domain 3	Transcription	Information	2.32	99	100	99.6	98.5	ABE
d.50.1	dsRNA-binding domain-like	RNA binding, m/tr	Regulation	4.1	99.8	100	100	100	ABE
a.203.1	Putative anticodon-binding domain of alanyl-tRNA synthetase (AlaRS)	Other enzymes	Metabolism	2.22	99.3	100	99.6	99.2	ABE
a.211.1	HD-domain/PDEase-like	Other enzymes	Metabolism	3.52	98.3	100	98.7	97.7	ABE
c.73.1	Carbamate kinase-like	Amino acids m/tr	Metabolism	3.99	98.6	100	100	96.2	ABE
c.8.5	GroEL apical domain-like	Protein modification	Processes_IC	3.72	100	100	100.4	100	ABE
d.79.4	PurM N-terminal domain-like	Other enzymes	Metabolism	4.12	99.5	100	99.6	100	ABE
a.16.1	S15/NS1 RNA-binding domain	RNA binding, m/tr	Regulation	2.75	99.5	100	99.6	100	ABE
c.23.15	Ribosomal protein S2	Translation	Information	2.36	98.8	100	99.2	98.5	ABE
d.67.2	Arginyl-tRNA synthetase (ArgRS), N-terminal 'additional' domain	Translation	Information	2.1	95.5	85.4	99.6	92.5	ABE
d.67.1	ThrRS/AlaRS common domain	Translation	Information	3.45	99.8	100	100	100	ABE
d.47.1	Ribosomal L11/L12e N-terminal domain	Translation	Information	2.27	96.7	85.4	99.6	96.2	ABE
g.39.1	Glucocorticoid receptor-like (DNA-binding domain)	DNA-binding	Regulation	5.7	99.8	100	100	100	ABE
f.41.1	Preprotein translocase SecY subunit	Transport	Processes_IC	2.28	99.8	100	100	100	ABE
d.55.1	Ribosomal protein L22	Translation	Information	2.4	99.5	100	100	99.2	ABE
c.86.1	Phosphoglycerate kinase	Energy	Metabolism	2.2	99	100	100	97.7	ABE
b.44.1	EF-Tu/eEF-1alpha/eIF2-gamma C-terminal domain	Translation	Information	3.8	99.8	100	100	100	ABE
a.60.2	RuvA domain 2-like	DNA replication/repair	Information	3.15	91.2	81.3	100	79.7	ABE
a.60.7	5' to 3' exonuclease, C-terminal subdomain	DNA replication/repair	Information	2.81	99.5	100	100	99.2	ABE
a.156.1	S13-like H2TH domain	Translation	Information	3.06	99.3	100	100	98.5	ABE
c.22.1	Ribosomal protein L4	Translation	Information	2.4	99.5	100	100	99.2	ABE
d.140.1	Ribosomal protein S8	Translation	Information	2.22	98.6	100	100.4	95.5	ABE
d.142.2	DNA ligase/mRNA capping enzyme, catalytic domain	DNA replication/repair	Information	3.18	100	100	100.4	100	ABE
b.39.1	Ribosomal protein L14	Translation	Information	2.34	99.5	100	100	99.2	ABE
d.12.1	Ribosomal proteins S24e, L23 and L15e	Translation	Information	2.67	99.5	100	100	99.2	ABE
d.92.1	Metalloproteases ("zincins"), catalytic domain	Proteases	Processes_IC	5.84	98.3	87.5	100	100	ABE
c.21.1	Ribosomal protein L13	Translation	Information	2.38	99.5	100	100	99.2	ABE
d.181.1	Insert subdomain of RNA polymerase alpha subunit	DNA-binding	Regulation	2.34	99	97.9	100	98.5	ABE
d.53.1	Ribosomal protein S3 C-terminal domain	Translation	Information	2.1	98.6	100	100	96.2	ABE
d.51.1	Eukaryotic type KH-domain (KH-domain type I)	RNA processing	Information	4.58	99.5	100	99.6	100	ABE
c.116.1	alpha/beta knot	Unknown function	Other	4.34	98.1	100	100	94.7	ABE
d.41.4	Ribosomal protein L16p/L10e	Translation	Information	2.37	99.8	100	100	100	ABE
d.41.2	Nicotinate/Quinolinate PRTase N-terminal domain-like	Coenzyme m/tr	Metabolism	3.03	98.8	100	99.2	98.5	ABE
g.41.3	Zinc beta-ribbon	Unknown function	Other	3.93	99.8	100	100	100	ABE
c.7.1	PFL-like glycyl radical enzymes	Other enzymes	Metabolism	2.74	99	100	100	97.7	ABE
c.1.1	Triosephosphate isomerase (TIM)	Energy	Metabolism	2.16	97.9	100	100	94	ABE
d.115.1	YrdC/RibB	Coenzyme m/tr	Metabolism	3.1	99	100	100	97.7	ABE

f.17.1	F1F0 ATP synthase subunit C	Energy	Metabolism	2.98	99	97.9	99.2	100	ABE
e.24.1	Ribosomal protein L1	Translation	Information	2.53	99.5	100	100	99.2	ABE
c.78.1	Aspartate/ornithine carbamoyltransferase	Amino acids m/tr	Metabolism	4.06	98.8	95.8	100.4	97.7	ABE
d.66.1	Alpha-L RNA-binding motif	RNA binding, m/tr	Regulation	4.31	99.8	100	100	100	ABE
b.80.6	Stabilizer of iron transporter SufD	Transport	Processes_IC	1.57	55.1	100	68.2	15.8	ABE
d.68.2	EPT/RTPC-like	RNA processing	Information	3.09	99.5	100	99.6	100	ABE
c.1.11	Enolase C-terminal domain-like	Energy	Metabolism	3.41	99.5	100	100	99.2	ABE
a.182.1	GatB/YqeY motif	Unknown function	Other	2.43	93.1	93.8	98.7	83.5	ABE

***min_set* (70 FSFs)**

FSF	SCOP functional description	Functional subcategories	Functional categories	Mean of G	f_T	f_A	f_B	f_E	DS
c.95.1	Thiolase-like	Other enzymes	Metabolism	7.01	99.8	100	100	100	ABE
d.144.1	Protein kinase-like (PK-like)	Kinases/phosphatases	Regulation	8.19	99	100	98.7	100	ABE
d.14.1	Ribosomal protein S5 domain 2-like	Translation	Information	7.61	100	100	100	100	ABE
b.43.3	Translation proteins	Translation	Information	6.27	99.8	100	100	100	ABE
c.23.5	Flavoproteins	Redox	Metabolism	4.75	96.4	77.1	98.7	100	ABE
a.35.1	lambda repressor-like DNA-binding domains	DNA-binding	Regulation	5.52	99	100	100	97.7	ABE
d.153.1	N-terminal nucleophile aminohydrolases (Ntn hydrolases)	Other enzymes	Metabolism	6.05	99.8	100	100	100	ABE
d.37.1	CBS-domain	Transport	Processes_IC	6.78	99.5	100	100	99.2	ABE
c.3.1	FAD/NAD(P)-binding domain	Small molecule binding	General	9.05	100	100	100	100	ABE
a.6.1	Putative DNA-binding domain	DNA-binding	Regulation	4.57	99.5	100	100	99.2	ABE
f.39.1	Multidrug resistance efflux transporter EmrE	Ion m/tr	Processes_IC	6.45	99.3	97.9	99.6	100	ABE
a.4.5	Winged helix DNA-binding domain	DNA-binding	Regulation	10.23	100	100	100	100	ABE
d.81.1	Glyceraldehyde-3-phosphate dehydrogenase-like, C-terminal domain	Energy	Metabolism	5.97	99.8	100	100	100	ABE
c.68.1	Nucleotide-diphospho-sugar transferases	Transferases	Metabolism	8.03	99.8	100	100	100	ABE
c.30.1	PreATP-grasp domain	Coenzyme m/tr	Metabolism	5.56	100	100	100	100	ABE
c.36.1	Thiamin diphosphate-binding fold (THDP-binding)	Small molecule binding	General	7.33	100	100	100	100	ABE
c.66.1	S-adenosyl-L-methionine-dependent methyltransferases	Transferases	Metabolism	9.87	100	100	100	100	ABE
b.92.1	Composite domain of metallo-dependent hydrolases	Other enzymes	Metabolism	4.94	98.3	91.7	100	98.5	ABE
d.58.11	EF-G C-terminal domain-like	Translation	Information	5.44	99.8	100	100	100	ABE
d.58.19	Bacterial exopeptidase dimerisation domain	Proteases	Processes_IC	4.07	98.8	91.7	100	100	ABE
d.58.18	ACT-like	Amino acids m/tr	Metabolism	5.04	99.5	100	100	99.2	ABE
d.104.1	Class II aaRS and biotin synthetases	Coenzyme m/tr	Metabolism	6.56	100	100	100	100	ABE
c.55.1	Actin-like ATPase domain	Other enzymes	Metabolism	8.72	99.8	100	100	100	ABE
c.55.3	Ribonuclease H-like	Nucleotide m/tr	Metabolism	7.58	99.8	100	100	100	ABE
d.127.1	Creatinase/aminopeptidase	Proteases	Processes_IC	4.39	100	100	100	100	ABE
c.61.1	PRTase-like	Nucleotide m/tr	Metabolism	5.76	100	100	100	100	ABE
c.94.1	Periplasmic binding protein-like II	Ion m/tr	Processes_IC	8.17	100	100	100	100	ABE
d.157.1	Metallo-hydrolase/oxidoreductase	Redox	Metabolism	6.43	100	100	100	100	ABE
d.159.1	Metallo-dependent phosphatases	Proteases	Processes_IC	6.59	99.8	100	100	100	ABE
e.8.1	DNA/RNA polymerases	DNA replication/repair	Information	5.11	99.8	100	100	100	ABE
a.96.1	DNA-glycosylase	DNA replication/repair	Information	3.81	99.8	100	100	100	ABE

c.58.1	Aminoacid dehydrogenase-like, N-terminal domain	Redox	Metabolism	4.71	100	100	100	100	ABE
b.38.1	Sm-like ribonucleoproteins	Translation	Information	4.84	98.8	100	98.3	100	ABE
c.56.5	Zn-dependent exopeptidases	Proteases	Processes_IC	6.6	100	100	100	100	ABE
d.265.1	Pseudouridine synthase	Nucleotide m/tr	Metabolism	5.23	99.5	100	99.6	100	ABE
c.67.1	PLP-dependent transferases	Transferases	Metabolism	8.59	100	100	100	100	ABE
c.26.2	Adenine nucleotide alpha hydrolases-like	Nucleotide m/tr	Metabolism	7.12	99.8	100	100	100	ABE
c.26.1	Nucleotidylyl transferase	Nucleotide m/tr	Metabolism	7.3	100	100	100	100	ABE
d.26.1	FKBP-like	Protein modification	Processes_IC	5.12	99.8	100	100	100	ABE
d.108.1	Acyl-CoA N-acyltransferases (Nat)	Transferases	Metabolism	7.8	99.8	100	100	100	ABE
c.79.1	Tryptophan synthase beta subunit-like PLP-dependent enzymes	Amino acids m/tr	Metabolism	5	99.5	100	99.6	100	ABE
c.23.12	Formate/glycerate dehydrogenase catalytic domain-like	Redox	Metabolism	4.84	100	100	100	100	ABE
c.23.16	Class I glutamine amidotransferase-like	Transferases	Metabolism	6.47	100	100	100	100	ABE
b.81.1	Trimeric LpxA-like enzymes	Other enzymes	Metabolism	5.34	99.8	100	100	100	ABE
c.108.1	HAD-like	Other enzymes	Metabolism	7.92	99.8	100	100	100	ABE
d.163.1	DNA breaking-rejoining enzymes	DNA replication/repair	Information	4.36	97.4	85.4	100	97.7	ABE
c.84.1	Phosphoglucomutase, first 3 domains	Carbohydrate m/tr	Metabolism	5.45	99.5	100	100	99.2	ABE
d.113.1	Nudix	DNA replication/repair	Information	5.74	99.5	97.9	100	100	ABE
d.122.1	ATPase domain of HSP90 chaperone/DNA topoisomerase II/histidine kinase	Protein modification	Processes_IC	7.76	100	100	100	100	ABE
a.128.1	Terpenoid synthases	Secondary metabolism	Metabolism	3.94	99.5	100	99.6	100	ABE
b.40.4	Nucleic acid-binding proteins	DNA replication/repair	Information	8.63	100	100	100	100	ABE
b.34.5	Translation proteins SH3-like domain	Translation	Information	4.47	99.8	100	100	100	ABE
d.218.1	Nucleotidyltransferase	Nucleotide m/tr	Metabolism	5.32	99.8	100	100	100	ABE
d.142.1	Glutathione synthetase ATP-binding domain-like	Coenzyme m/tr	Metabolism	6.86	100	100	100	100	ABE
c.2.1	NAD(P)-binding Rossmann-fold domains	Small molecule binding	General	11.35	99.8	100	100	100	ABE
c.1.28	Radical SAM enzymes	Other enzymes	Metabolism	6.38	99.5	100	99.6	100	ABE
f.38.1	MFS general substrate transporter	Ion m/tr	Processes_IC	9	99.8	100	100	100	ABE
c.31.1	DHS-like NAD/FAD-binding domain	Other enzymes	Metabolism	5.19	99.8	100	100	100	ABE
c.37.1	P-loop containing nucleoside triphosphate hydrolases	Small molecule binding	General	13.78	100	100	100	100	ABE
a.27.1	Anticodon-binding domain of a subclass of class I aminoacyl-tRNA synthetases	Translation	Information	5.27	100	100	100	100	ABE
c.1.2	Ribulose-phoshate binding barrel	Nucleotide m/tr	Metabolism	4.93	100	100	100	100	ABE
c.1.4	FMN-linked oxidoreductases	Protein interaction	General	5.6	99.8	100	100	100	ABE
c.1.9	Metallo-dependent hydrolases	Other enzymes	Metabolism	6.16	99.8	100	100	100	ABE
c.124.1	NagB/RpiA/CoA transferase-like	Transferases	Metabolism	5.45	99.8	100	100	100	ABE
c.72.1	Ribokinase-like	Other enzymes	Metabolism	5.16	99.8	100	100	100	ABE
d.128.1	Glutamine synthetase/guanido kinase	Amino acids m/tr	Metabolism	3.86	99.8	100	100	100	ABE
a.100.1	6-phosphogluconate dehydrogenase C-terminal domain-like	Energy	Metabolism	5.93	99.8	100	100	100	ABE
c.1.12	Phosphoenolpyruvate/pyruvate domain	Energy	Metabolism	4.8	99.8	100	100	99.2	ABE
c.1.10	Aldolase	Carbohydrate m/tr	Metabolism	6.37	99.8	100	100	100	ABE
c.87.1	UDP-Glycosyltransferase/glycogen phosphorylase	Polysaccharide m/tr	Metabolism	7.49	100	100	100	100	ABE

Table S2. FSF molecular functions. The 1,416 FSFs (*total_set*) that encompass the *352_set*, and the urancestral *max_set* and *min_set* (*see* Supplementary Table 1) were assigned to 7 major categories and 49 sub-categories of molecular functions. Note that 4 FSFs in the set of 1,416 FSFs and 2 FSFs in the *352_set* did not belong to any functional category.

Major categories	Sub-categories	No. FSFs in *min_set*	No. FSFs in *max_set*	No. FSFs in *352_set*	No. FSFs in *total_set*
General	Protein interaction	1	1	2	33
	Small molecule binding	4	4	5	22
	Ion binding	0	0	2	10
	Lipid/membrane binding	0	0	0	2
	Ligand binding	0	0	1	3
	General	0	0	3	28
	Structural protein	0	0	0	3
Information	DNA replication/repair	5	10	19	64
	Translation	6	34	52	89
	Chromatin structure	0	0	0	6
	Transcription	0	4	9	24
	RNA processing	0	2	5	9
	Nuclear structure	0	0	0	0
Metabolism	Coenzyme m/tr	3	6	26	50
	Amino acids m/tr	3	5	10	20
	Other enzymes	10	18	46	147
	Carbohydrate m/tr	2	3	7	30
	Transferases	6	6	9	29
	Polysaccharide m/tr	1	1	1	20
	Redox	4	4	21	53
	Secondary metabolism	1	2	4	10
	Energy	3	9	13	53
	Nucleotide m/tr	7	8	21	29
	Photosynthesis	0	0	0	18
	E- transfer	0	0	4	28
	Nitrogen m/tr	0	0	1	1
	Storage	0	0	0	0
	Lipid m/tr	0	0	1	14
	Cell envelope m/tr	0	0	0	4
Intra-cellular Processes	Protein modification	2	6	7	32
	Transport	1	4	12	48
	Proteases	4	5	7	51
	Ion m/tr	3	3	6	21
	Cell cycle, Apoptosis	0	0	0	18
	Phospholipid m/tr	0	0	0	6
	Cell motility	0	0	0	16

	Trafficking/secretion	0	0	0	0
Extra-cellular Processes	Cell adhesion	0	0	1	30
	Immune response	0	0	0	13
	Blood clotting	0	0	0	3
	Toxins/defense	0	0	0	23
Regulatoin	Kinases/phosphatases	1	2	4	13
	DNA-binding	3	5	14	61
	RNA binding, m/tr	0	3	7	17
	Signal transduction	0	0	4	51
	Other regulatory function	0	1	2	30
	Receptor activity	0	1	1	12
Others	Unknown function	0	5	23	144
	Viral proteins	0	0	0	28

Table S3. Functional enrichment. We examined the enrichment or depletion of functional sub-categories in the urancestral *min_set* and *max_set* FSFs (*see* Supplementary Tables 1 and 2). *Nuclear structure*, *storage*, and *trafficking/secretion* had no FSFs in the 420 FL genomes we analyzed and were excluded in the analysis. The *sample* (*min_set* or the *max_set*) was compared to the *background* (*bkg_set* with 1,416 FSFs) to test the statistical significance of a particular sub-category. The statistical strength of the enrichment or depletion was evaluated using *P* values that were calculated based on the hypergeometric distribution.

min_set* versus *bkg_set								
Function	FSFs *min_set*	Total no. *min_set*	FSFs *bkg_set*	Total no. *bkg_set*	Rate in *min_set*	Rate in *bkg_set*	Ratio	*P*
Coenzyme m/tr, Metabolism	3	70	50	1416	0.043	0.035	1.213	0.45
DNA replication/repair, Information	5	70	64	1416	0.071	0.045	1.580	0.2
Protein modification, Processes_IC	2	70	32	1416	0.029	0.023	1.264	0.48
Amino acids m/tr, Metabolism	3	70	20	1416	0.043	0.014	3.034	0.077
Other enzymes, Metabolism	10	70	147	1416	0.143	0.104	1.376	0.18
Protein interaction, General	1	70	33	1416	0.014	0.023	0.613	0.51
Carbohydrate m/tr, Metabolism	2	70	30	1416	0.029	0.021	1.349	0.43
Kinases/phosphatases, Regulation	1	70	13	1416	0.014	0.009	1.556	0.49
Transferases, Metabolism	6	70	29	1416	0.086	0.021	4.185	0.0021
Transport, Processes_IC	1	70	48	1416	0.014	0.034	0.421	0.3
Polysaccharide m/tr, Metabolism	1	70	20	1416	0.014	0.014	1.011	0.65
Translation, Information	6	70	89	1416	0.086	0.063	1.364	0.28
Redox, Metabolism	4	70	53	1416	0.057	0.037	1.527	0.26
Secondary metabolism, Metabolism	1	70	10	1416	0.014	0.007	2.023	0.41
Small molecule binding, General	4	70	22	1416	0.057	0.015	3.679	0.021
Proteases, Processes_IC	4	70	51	1416	0.057	0.036	1.587	0.25
DNA-binding, Regulation	3	70	61	1416	0.043	0.043	0.995	0.66
Energy, Metabolism	3	70	53	1416	0.043	0.037	1.145	0.5
Ion m/tr. Processes_IC	3	70	21	1416	0.043	0.015	2.890	0.081
Nucleotide m/tr. Metabolism	7	70	29	1416	0.1	0.020	4.883	0.00037
Photosynthesis, Metabolism	0	70	18	1416	0	0.013	0	0.4
Ion binding, General	0	70	10	1416	0	0.007	0	0.6
Lipid/membrane binding, General	0	70	2	1416	0	0.001	0	0.91
Ligand binding, General	0	70	3	1416	0	0.002	0	0.85
General, General	0	70	28	1416	0	0.020	0	0.24
Structural protein, General	0	70	3	1416	0	0.002	0	0.85
Chromatin structure, Information	0	70	6	1416	0	0.004	0	0.73
Transcription, Information	0	70	24	1416	0	0.017	0	0.29
RNA processing, Information	0	70	9	1416	0	0.006	0	0.62
E- transfer, Metabolism	0	70	28	1416	0	0.020	0	0.24
Nitrogen m/tr, Metabolism	0	70	1	1416	0	0.001	0	0.96
Lipid m/tr, Metabolism	0	70	14	1416	0	0.010	0	0.49
Cell envelope m/tr, Metabolism	0	70	4	1416	0	0.003	0	0.82
Unknown function, Other	0	70	144	1416	0	0.102	0	0.00045
Viral proteins, Other	0	70	28	1416	0	0.020	0	0.24

Cell adhesion, Processes_EC	0	70	30	1416	0	0.021	0	0.22
Immune response, Processes_EC	0	70	13	1416	0	0.009	0	0.53
Blood clotting, Processes_EC	0	70	3	1416	0	0.002	0	0.85
Toxins/defense, Processes_EC	0	70	23	1416	0	0.016	0	0.31
Cell cycle, Apoptosis, Processes_IC	0	70	18	1416	0	0.013	0	0.4
Phospholipid m/tr, Processes_IC	0	70	6	1416	0	0.004	0	0.73
Cell motility, Processes_IC	0	70	16	1416	0	0.011	0	0.45
RNA binding, m/tr, Regulation	0	70	17	1416	0	0.012	0	0.4
Signal transduction, Regulation	0	70	51	1416	0	0.036	0	0.073
Other regulatory function, Regulation	0	70	30	1416	0	0.021	0	0.22
Receptor activity, Regulation	0	70	12	1416	0	0.008	0	0.55

max_set* versus *bkg_set

Molecular function	FSF *max_set*	Total no. *max_set*	FSFs *bkg_set*	Total no. *bkg_set*	Rate in *max_set*	Rate in *bkg_set*	Ratio	*P*
Coenzyme m/tr, Metabolism	6	152	50	1416	0.039	0.035	1.118	0.43
Receptor activity, Regulation	1	152	12	1416	0.006	0.008	0.776	0.6
DNA-binding, Regulation	5	152	61	1416	0.033	0.043	0.764	0.33
Redox, Metabolism	4	152	53	1416	0.026	0.037	0.703	0.3
RNA binding, m/tr, Regulation	3	152	17	1416	0.020	0.012	1.644	0.26
Transcription, Information	4	152	24	1416	0.026	0.017	1.553	0.24
Proteases, Processes_IC	5	152	51	1416	0.033	0.036	0.913	0.52
Ion m/tr, Processes_IC	3	152	21	1416	0.020	0.015	1.331	0.37
Protein modification, Processes_IC	6	152	32	1416	0.040	0.023	1.747	0.11
Other enzymes, Metabolism	18	152	147	1416	0.118	0.104	1.141	0.28
Protein interaction, General	1	152	33	1416	0.007	0.023	0.283	0.11
Carbohydrate m/tr, Metabolism	3	152	30	1416	0.020	0.021	0.932	0.58
Transport, Processes_IC	4	152	48	1416	0.026	0.034	0.776	0.38
Unknown function, Other	5	152	144	1416	0.033	0.102	0.323	0.00081
Translation, Information	34	152	89	1416	0.224	0.063	3.559	1E-12
Energy, Metabolism	9	152	53	1416	0.060	0.037	1.582	0.1
Transferases, Metabolism	6	152	29	1416	0.039	0.020	1.927	0.078
Polysaccharide m/tr, Metabolism	1	152	20	1416	0.007	0.014	0.466	0.34
Other regulatory function, Regulation	1	152	30	1416	0.007	0.021	0.310	0.15
Secondary metabolism, Metabolism	2	152	10	1416	0.013	0.007	1.863	0.27
Small molecule binding, General	4	152	22	1416	0.026	0.015	1.694	0.19
Amino acids m/tr, Metabolism	5	152	20	1416	0.033	0.014	2.329	0.052
Nucleotide m/tr, Metabolism	8	152	29	1416	0.053	0.020	2.570	0.0083
DNA replication/repair, Information	10	152	64	1416	0.066	0.045	1.456	0.13
RNA processing, Information	2	152	9	1416	0.013	0.006	2.070	0.24
Kinases/phosphatases, Regulation	2	152	13	1416	0.013	0.009	1.433	0.41
Photosynthesis, Metabolism	0	152	18	1416	0	0.013	0	0.12

Ion binding, General	0	152	10	1416	0	0.007	0	0.31
Lipid/membrane binding, General	0	152	2	1416	0	0.001	0	0.81
Ligand binding, General	0	152	3	1416	0	0.002	0	0.69
General, General	0	152	28	1416	0	0.020	0	0.04
Structural protein, General	0	152	3	1416	0	0.002	0	0.69
Chromatin structure, Information	0	152	6	1416	0	0.004	0	0.5
E- transfer, Metabolism	0	152	28	1416	0	0.020	0	0.04
Nitrogen m/tr, Metabolism	0	152	1	1416	0	0.001	0	0.88
Lipid m/tr, Metabolism	0	152	14	1416	0	0.010	0	0.19
Cell envelope m/tr, Metabolism	0	152	4	1416	0	0.003	0	0.62
Viral proteins, Other	0	152	28	1416	0	0.020	0	0.04
Cell adhesion, Processes_EC	0	152	30	1416	0	0.021	0	0.031
Immune response, Processes_EC	0	152	13	1416	0	0.009	0	0.22
Blood clotting, Processes_EC	0	152	3	1416	0	0.002	0	0.69
Toxins/defense, Processes_EC	0	152	23	1416	0	0.016	0	0.069
Cell cycle, Apoptosis, Processes_IC	0	152	18	1416	0	0.013	0	0.12
Phospholipid m/tr, Processes_IC	0	152	6	1416	0	0.004	0	0.5
Cell motility, Processes_IC	0	152	16	1416	0	0.011	0	0.16
Signal transduction, Regulation	0	152	51	1416	0	0.036	0	0.0026
